# Nutrient complexity triggers transitions between solitary and colonial growth in bacterial populations

**DOI:** 10.1038/s41396-021-00953-7

**Published:** 2021-03-17

**Authors:** Glen G. D’Souza, Vanessa R. Povolo, Johannes M. Keegstra, Roman Stocker, Martin Ackermann

**Affiliations:** 1 Department of Environmental Systems Sciences, Microbial Systems Ecology Group, Institute of Biogeochemistry and Pollutant Dynamics, ETH-Zurich, Zurich, Switzerland; 2 Department of Environmental Microbiology, Eawag: Swiss Federal Institute of Aquatic Sciences, Duebendorf, Switzerland; 3 Department of Civil, Environmental and Geomatic Engineering, Institute of Environmental Engineering, ETH Zurich, Zurich, Switzerland

**Keywords:** Microbial ecology, Water microbiology, Biofilms

## Abstract

Microbial populations often experience fluctuations in nutrient complexity in their natural environment such as between high molecular weight polysaccharides and simple monosaccharides. However, it is unclear if cells can adopt growth behaviors that allow individuals to optimally respond to differences in nutrient complexity. Here, we directly control nutrient complexity and use quantitative single-cell analysis to study the growth dynamics of individuals within populations of the aquatic bacterium *Caulobacter crescentus*. We show that cells form clonal microcolonies when growing on the polysaccharide xylan, which is abundant in nature and degraded using extracellular cell-linked enzymes; and disperse to solitary growth modes when the corresponding monosaccharide xylose becomes available or nutrients are exhausted. We find that the cellular density required to achieve maximal growth rates is four-fold higher on xylan than on xylose, indicating that aggregating is advantageous on polysaccharides. When collectives on xylan are transitioned to xylose, cells start dispersing, indicating that colony formation is no longer beneficial and solitary behaviors might serve to reduce intercellular competition. Our study demonstrates that cells can dynamically tune their behaviors when nutrient complexity fluctuates, elucidates the quantitative advantages of distinct growth behaviors for individual cells and indicates why collective growth modes are prevalent in microbial populations.

## Introduction

Bacteria in natural environments exhibit distinct behavioral states such as living in close spatial proximities of each other within surface-attached biofilms or in solitary planktonic states [1–4]. Transitions between aggregation and planktonic behaviors are common and are governed by diverse molecular cues [4]. However, the functionality of distinct behavioral modes, which in all likelihood represent adaptations to different environmental conditions, remains understudied and unclear. This is because it is challenging to identify the benefits that collective or solitary growth modes provide to individual cells and analyze when it is advantageous for cells to switch between growth modes. One explanation could be that the benefit of different growth modes depends strongly on the nutrient environment, since nutrients fundamentally influence cellular pathways that drive growth. Therefore, we asked if the complexity of the growth substrate determines whether cells engage in solitary or aggregative behaviors.

Aggregative growth, where individuals self-organize in close spatial association, can be beneficial when cells release compounds that modify the extracellular environment. Prominent examples of such compounds include iron-chelators [5] and enzymes that degrade complex polysaccharides like chitin [6] and disaccharides like sucrose [7]. These compounds generate diffusible resources – for example, simple sugars or metals in a bioavailable form – that are transiently accessible for all nearby cells. Cells in close spatial associations can thus benefit from diffusible resources emerging from the activity of other individuals [1, 5, 8]. In contrast, solitary behavior, where cells are planktonic and often motile, reduces local competition and allows dispersal to new habitats [2, 4]. This suggests that growth substrates can be key modulators of behavior in bacteria. An important question thus arises: How does the complexity of the nutrient affect dynamic behavioral transitions in bacteria?

Nutrient environments that bacteria encounter in natural ecosystems are dominated by complex polysaccharides [9–12]. These generally have large molecular sizes and are often particulate. Thus, cells must degrade these polymeric substrates using extracellular enzymes into simpler mono- and multi-meric forms, which can then be taken up by cells and catabolized [13]. In well-mixed environments, extracellular degradation products generated by the enzymatic machinery of an individual cell can be lost due to diffusion [5]. As a result, polymeric growth substrates are expected to reduce the productivity of microbial populations relative to monomeric substrates. Therefore, we reasoned, in accordance with previous work on yeast [7], that growth on complex carbohydrates should favor growth of cells as collectives. This is because group behavior will lead to an increase in the per capita payoff due to the reduction in diffusional loss and higher benefit from the degradative activities of neighboring cells. Our goal here was to obtain a quantitative estimate of the advantages of aggregation at the level of individual cells and to understand when it is beneficial for cells to switch from aggregative to planktonic growth.

We used *Caulobacter crescentus* as a model system to study growth behaviors of cells on xylan, a naturally abundant polysaccharide. Xylan is a major component of plant biomass (up to 30%) and thus is a common recalcitrant compound in natural ecosystems [14]. *C. crescentus* is ubiquitous in both aquatic and terrestrial environments, and has the biochemical repertoire to metabolize xylan in addition to several other complex polysaccharides [12, 15–17]. Asymmetrical cell division in *C. crescentus* gives rise to two different cell types, a sessile stalked cell and a flagellated swarmer cell. The swarmer cell is motile and differentiates into a sessile stalked cell before initiating cell division [18]. The differentiation between motile swarmer cells and sessile stalked cells is controlled by nutrient signals [19] and thereby, *C. crescentus* represents a good model system to study behavioral responses to nutrient complexity in bacteria.

**Fig. 1:**
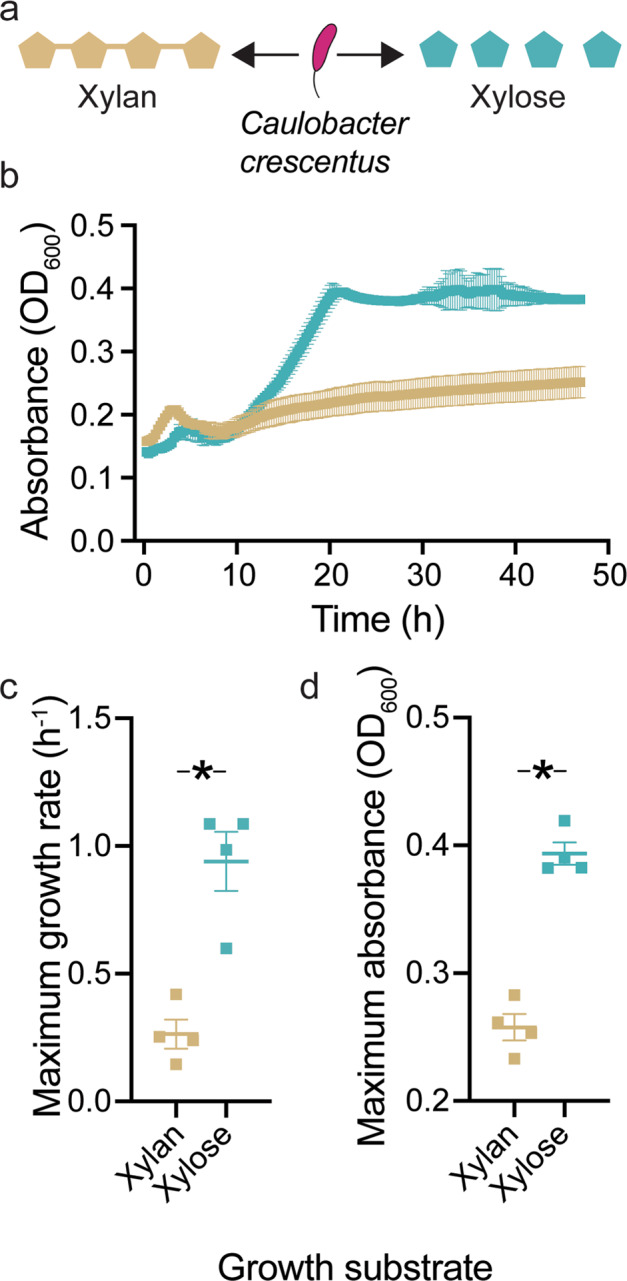
The polymer xylan limits the growth of *C. crescentus* compared to the monomer xylose. **a***Caulobacter crescentus* CB15 cells were grown in the same concentration (%weight/volume) of the polymer 0.05% xylan or its constituent monomer 0.05% xylose and **b** the growth dynamics of populations (optical density at 600 nm) were measured. **c** Maximum growth rate and **d** maximum optical density observed over the course of a growth cycle. Compared to populations on xylan, populations grown on xylose achieve higher growth rates (h^−1^) (independent samples *t*-test, *P* = 0.0019, *R*^2^ (eta^2^) = 0.82, *n*_populations_ = 4 for each treatment) and greater maximum optical density (independent samples *t*-test, *P* = 0.0001, *R*^2^ (eta^2^) = 0.94, *n*_*populations*_ = 4 for each treatment). Squares, horizontal lines and whiskers indicate the individual measurements for each biological replicate population (*n*_populations_ = 4), the mean and the 95% confidence interval (CI), respectively, on xylan (yellow) and xylose (blue). Asterisks indicate significant differences.

We used well-mixed batch cultures to study the growth dynamics of *C. crescentus* populations and combined this with a quantitative analysis of behaviors at the single-cell level in the presence of either the polysaccharide xylan or its constituent monosaccharide xylose (Fig. 1a). In order to track and quantify the growth and behavior of individual cells, we used a combination of microfluidics, time-lapse microscopy and automated image analysis. These measurements were performed in microfluidic devices containing micron-scale growth chambers (Supplementary Fig. 1), where bacterial cells could grow and move, and nutrients could freely diffuse, albeit at a reduced rate compared to well-mixed conditions [20]. The environment that cells experienced within chambers could be controlled and altered by changing the nutrient source from polymers to monomers and vice-versa, making it possible to measure behavioral transitions at the level of individual cells.

## Materials and methods

### Bacterial strains, media and growth assays

We used the wildtype (WT) strain *Caulobacter crescentus* CB15 (mKate2: GD2 and Venus: GD3) and *C. crescentus* NA1000 (mKate2: AKS295) strain variants that contained chromosomally incorporated phenotypic markers: fluorescent proteins mKate2 or Venus under a constitutive pLac promoter for most experiments [21]. Strains were cultured in Peptone Yeast Extract Broth [22] (PYE-B) and grown for 30 h at 30 °C. Cells from these cultures were used for growth experiments in M2 minimal medium [16] containing either xylan (Megazyme, Ireland) or xylose (Sigma Aldrich, Switzerland). Carbon sources were prepared using nanopure water and filter sterilized using 0.40 μm Surfactant-Free Cellulose Acetate filters (Corning, USA). Concentrations for batch experiments ranged from 0.01–0.1% (weight/volume) for batch experiments and 0.05% (weight/volume) for microfluidic experiments for both xylan and xylose. Well-mixed batch experiments in xylan or xylose media were performed in 96-well plates and growth dynamics was measured using a micro-well plate reader (Biotek, USA). See Supplementary Methods for detailed information about growth conditions and media recipes.

### Microfluidics and time-lapse microscopy

Microfluidics experiments were performed as described previously [20, 22–24]. Cell growth and behavior was imaged within chambers which were 60 × 60 × 0.56 μm (length × width × height; Supplementary Fig. 1). Within these chambers, cells could bond to the glass surface and experienced the medium that the diffused through the lateral flow channels. Microscopy imaging was performed using either an Olympus IX81 or IX83 inverted microscope system (Olympus, Japan) with automated stage controller (Marzhauser Wetzlar, Germany), shutter, and laser-based autofocus system (Olympus ZDC 1 and 2). Chambers were imaged in parallel on the same PDMS chip, and phase-contrast and fluorescent (mKate2 and/or Venus) images of each position were taken every 1 or 8 min. The microscopy units and PDMS chip was maintained at 30 °C using a cellVivo microscope incubation system (Pecon GmbH, Germany) or a Cube incubation system (Life Imaging Services, Switzerland). See Supplementary Methods for detailed information about preparation of microfluidic growth chambers and setup of the experiment. Nutrient switches were performed by disconnecting the larger tubing from the syringe and reconnecting it to a different syringe containing the new carbon source. All experiments were run at a flow rate of 0.1 ml h^−1^ and flow velocity 400 mm h^−1^, which ensures nutrients enter the chamber through diffusion. Mock nutrient switches for the corresponding negative controls such as ‘xylan to xylan’ and ‘xylose to xylose’ were also performed in order to exclude the possibility that dispersal was due to a change in flow conditions or other physical artefacts introduced due to the switching process. Cells experienced the new nutrient condition ~20 min after the switch.

### Swimming assays

Cells were inoculated into chambers fed with 0.05% xylan or 0.05% xylose and then imaged for 4  with a high frame rate (7.5 frames s^−1^). Only the swimming speeds of motile swarmer cells were computed and sessile cells were ignored.

### Image analysis

Image processing was performed in Matlab v2017b and newer in combination with ilastik v1.2 and newer [25]; and/or SuperSegger [26]. Only fluorescence channel images were used for alignment, segmentation, tracking and linking. Images were cropped at the boundaries of each microfluidic chamber. Growth properties and spatial locations were directly derived from the downstream processing tools of SuperSegger (gateTool and superSeggerViewer) and ilastik (Tracking plugin). Spatial distances between cells were computed from segmentation data using the R package *spatstat* [27]. Lineage trees were reconstructed using a custom R script in combination with the *rgl* package in R. To calculate swimming speeds, spatial coordinates of individual cells from time-lapse images captured with a high frame rate (7.5 frames s^−1^) were manually mapped with ImageJ. The trajectories and swimming speed of individual cells were then computed using the *traj* package in R [28]. Using the cell coordinates obtained from segmentation and tracking, spatial densities in each chamber before and after switching nutrients were computed by dividing the number of bacteria by the area of the smallest rectangle encompassing the cells in every image. Single solitary cells far away from a colony, usually located at the chamber edge, were excluded from the analysis. Time series were aligned to the time of the nutrient switch and normalized by the density 4 h before the switch.

### Datasets and statistical analysis

All batch experiments were replicated four times. Growth curves were analyzed in R using the *growthcurver* package [29] and GraphPad Prism v8 (GraphPad Software, USA). The microscopy dataset set consists of nine chambers each for xylose and xylan; and 6 chambers each for xylose and xylan. These are grouped into three biological replicates wherein each biological replicate is fed by media through one unique channel in a microfluidic chip and a total of three chambers fed by the same channel. Cells with negative growth rates (*C. crescentus* CB15: 40% for xylan, 32% xylose, 36% for xylan and xylose; *C. crescentus* NA1000: 57% for xylan, 64% xylose) were excluded from the analysis after visual curation, and represent artefacts, mistakes in linking during the segmentation or tracking process or non-growing deformed cells. For *C. crescentus* CB15, 2197 cells were analyzed for xylose,11867 cells were analyzed for xylan, 2218 cells were analyzed for xylose and xylan. Each chamber was treated as an independent sample. Each figure depicts means or medians of nine chambers for each condition, i.e., xylan or xylose. Non-linear regression models in GraphPad Prism v 8.0 (GraphPad Software, USA) were applied to determine relationships between independent measures such as: number of cells versus time, growth rate versus cell birth time, growth rate versus number of cells, and times to reach half maximum optical density versus initial cell density. The best regression model that fits the data was selected based on the highest R^2^ or eta^2^ value. Detailed equations for non-linear regression models are shown in Supplementary Methods. Comparisons were considered statistically significant when *P* < *0.05* or when False Discovery Rate (FDR) corrected *Q* < *0.05*. FDR corrections were applied when multiple *t* tests were performed for the same dataset. Measures of effect size are represented by the R^2^ or eta^2^ value. All statistical analyses were performed in GraphPad Prism v 8.0 (GraphPad Software, USA) or SPSS statistics v23 (IBM, USA) and Rstudio v1.1.463 (Rstudio inc).

### Viscosity measurements

Rheology measurements were performed on a double-wall cuvette rheometer (Anton Paar) at 25 °C. The reported viscosity value is the mean and standard deviation of the viscosity averaged over the shear rate range 1–300 s^−1^.

## Results

### The polysaccharide xylan limits the growth of *C. crescentus* cells compared to the monomer xylose in well-mixed environments

We first tested our hypothesis that in well-mixed conditions the polymer xylan would limit the productivity of microbial populations relative to the monomer xylose. To determine if this was the case, we grew *C. crescentus* cells in the same concentration (weight/volume) of either the polymer xylan or its monomeric constituent xylose, both provided as the sole carbon source (Fig. 1a). We then compared the maximum growth rate and the maximal population size over the course of a 54 h growth cycle (Fig. 1b). In line with expectations, populations growing on the monomer xylose achieved higher growth rates and a higher maximum population size (Fig. 1b–d). This was true for all concentrations (0.01–0.1%) of monomer and polymer tested (Supplementary Fig. 2). These findings suggest that in well-mixed environments of equal carbon concentration, the complexity of the growth substrate governs the growth of *C. crescentus* populations.

### Cells engage in colonial behaviors on xylan whereas they exhibit solitary behaviors on xylose

Group formation could be a key mechanism through which cells could overcome polymer-induced growth limitations that exist in well-mixed environments. Collective behavior would allow cells to increase their local cell density, which leads to higher local concentrations of the monomeric products of polymer degradation. To test this prediction, we tested whether xylose and xylan elicit different behavioral responses in *C. crescentus*. We used microfluidic growth chambers in which cells were forced to grow as a monolayer. Our expectation was that growth within these devices would provide the spatial structure to overcome the growth limitations observed in well-mixed conditions (Supplementary Fig. 1). We tracked and quantified movement, and growth of individual cells using time-lapse microscopy and image analysis. Chambers were constantly replenished with minimal medium containing either xylose or xylan through a main nutrient feeding channel, as described elsewhere [20, 23, 24].

**Fig. 2:**
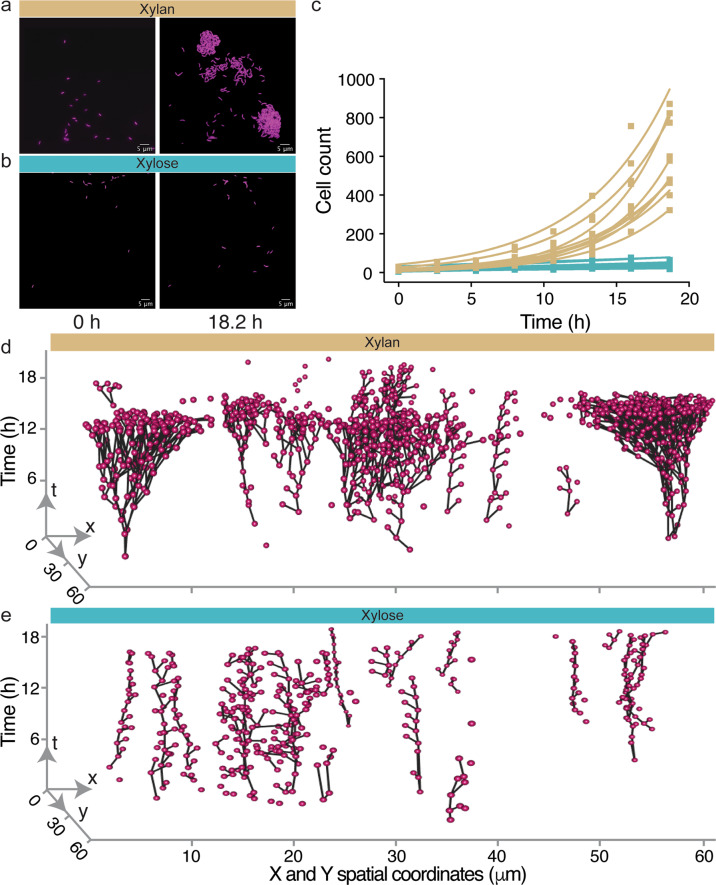
Cells display solitary behavior on xylose and aggregative behavior on xylan. Representative images of *C. crescentus* CB15 cells (labeled with constitutively expressed mKate2, false colored as magenta) at different time points within the microfluidic growth chambers supplied with either xylan (**a**) or xylose (**b***)* as the sole source of carbon. **c** On xylan (yellow), the number of sessile cells in the growth chamber increases with time, whereas on xylose (blue) it remains nearly constant. Squares indicate the number of cells present at a given time point in each chamber (*n*_chambers_ = 9), with a linear or exponential regression line for each chamber (xylose, linear regression model, *R*^2^ = 0.69–0.92, slope = 1.22–3.27, *P* < 0.01; xylan, exponential growth model, *R*^2^ = 0.92–0.99, doubling time = 2.89–4.15 h). Lineage trees reconstructed from time-lapse images of cells within one chamber, for xylan (**d**) and xylose (**e**). Cells (magenta spheres) are plotted as a function of their spatial location (*x* and *y* axes) and time *t*. Black lines connect cells that are related through cell division, and branching points mark division events. Representative time-lapse images of cells in xylan and xylose are shown in Supplementary Videos 1 and 2, respectively. Additional lineage trees from the other chambers are depicted in Supplementary Figs. 3 and 4 and a visual representation of lineage development in one representative xylan-fed chamber is shown in Supplementary Video 3.

We found that *C. crescentus* displayed strikingly disparate behaviors in xylan and xylose: cells formed microcolonies on the polymer xylan (Fig. 2a, Supplementary Video 1), whereas on the monomer xylose they did not (Fig. 2b, Supplementary Video 2). We analyzed the temporal dynamics of cell growth and movement in the two carbon sources by following individual cells using cell segmentation and tracking. Mapping the lineages based on division events for all the cells in a chamber revealed that the microcolonies on the polymer xylan originated from a single progenitor cell (Fig. 2d, Supplementary Fig. 3a–c; Supplementary Video 3). This finding indicates that microcolonies were a result of swarmer cells not dispersing after division, rather than a product of secondary aggregation by planktonic cells. In contrast, in the monomer xylose only the stalked cells remained in the same position after cell division, whereas the presumably flagellated swarmer cells moved away (Fig. 2e, Supplementary Fig. 4a–c). As a consequence of this difference in behavior, the number of sessile cells increased much more rapidly in xylan. The number of cells in a growth chamber doubled on average every 3.6 ± 0.54 h in xylan (mean ± 95% CI, Fig. 2c) but took 15.50 ± 7.55 h to double in xylose (mean ± 95% CI, Fig. 2c). These differences occurred despite a similar propensity to produce offspring per sessile cell in the two substrates (Supplementary Fig. 5), and thus were driven by the reduced rate at which cells dispersed in xylan.

To understand how these behavioral differences influenced the spatial distribution of cells, we quantified the intercellular distance between cells in a growth chamber. Specifically, we measured the intercellular distances between a focal cell and its 10, 20, 30, 40, 50, 60, 70, 80, 90 and 100 nearest neighbors. This analysis revealed that the intercellular distance between *C. crescentus* cells was consistently lower in xylan compared to xylose regardless of the number of nearest neighbors considered (Supplementary Fig. 6). Thus, *C. crescentus* individuals display strikingly disparate behaviors on the polymer xylan and the monomer xylose, causing populations grown on xylan to have an increased local cell density and decreased spatial distances between individuals, compared to cells grown on xylose.

### Colonial growth behavior provides a benefit to cells growing on the polymer xylan through increased cell density

**Fig. 3:**
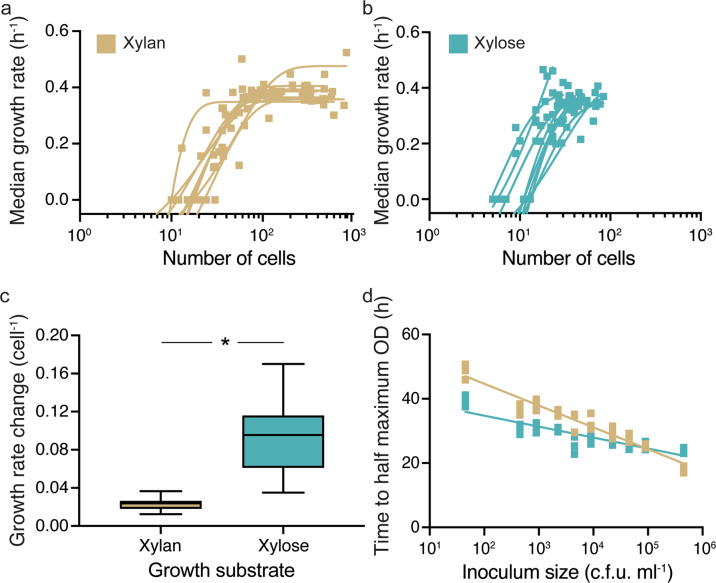
Aggregative behavior results in an increase in cell growth in xylan within microfluidic chambers. Median single cell growth rates (h^−1^) of *C. crescentus* CB15 cells as a function of the number of cells in a chamber on (**a**) xylan and (**b**) xylose. After binning cells based on their birth times (bins: 0–2.66 h, 2.67–5.33 h, 5.34–8 h, 8.1–10.66 h, 10.67–13.33 h, 13.34–16 h, 16.1–18.6 h) and hence the number of cells present during their growth, we determined which non-linear regression model can best predict (based on the *R*^2^ fit, see Methods and Supplementary Methods for detailed description) the relationship between median growth rate and cell number. Squares represent data for a single bin from one chamber (yellow: xylan, blue: xylose), and lines indicate the trajectory of growth rates for each chamber. In xylan (**a**), the relationship between median growth rate and the number of cells within a chamber was best explained by an exponential growth model (*n*_chambers_ = 9, *R*^2^ = 0.67–0.83). Maximum growth rates were reached when the number of cells chambers reached 40–110. In xylose (**b**), median growth rates also increased with the density in the chamber and were also best explained by an exponential growth model (*n*_chambers_ = 9, *R*^2^ = 0.72–84). An analysis of covariance further revealed that there are significant differences in growth rate with cellular density when birth-time is used as a covariate in the xylan environment (*F*_1,6_ = 12.37, *P* < 0.01, *R*^2^ = 0.56, eta^2^ = 0.49) but not the xylose environment (*F*_1,6_ = 2.12, *P* > 0.05, *R*^2^ = 0.24, eta^2^ = 0.18). **c** In xylan, on average a four-fold lower slope of growth rate with number of cells present in the chamber is observed compared to that in xylose (Mann–Whitney test, *P* = 0.0006, R^2^ (eta squared) = 0.76, *n*_*chambers*_ = 9 for each treatment). In **c**, box plots extend from the 25th to 75th percentiles and whiskers indicate the 10th (bottom) and 90th (top) percentiles of median growth rates. Asterisks indicate statistically significant differences between groups, respectively. Also see Supplementary Fig. 7c for a plot of change in growth rate per cell in xylan and xylose. **d** Initial cell density (cfu ml^−1^) has a stronger influence on time to reach half maximum optical density in xylan than in xylose, in well-mixed *C. crescentus* CB 15 populations. This is indicated by a higher slope of linear regression of the times to reach half maximum optical density on xylan compared to xylose (semi-log regression model, xylan: *R*^2^ = 0.93, slope = −6.78 h per 10 cells; xylose: *R*^2^ = 0.77, slope = −3.41 h per 10 cells; slopes differ significantly, *P* < 0.05, *n*_populations_ = 4). Squares indicate the measurements for each biological replicate (*n* = 4) and lines show the fit of the regression model. Also see Supplementary Fig. 7f for the relationship between growth rate and inoculum density.

In order to quantify the influence of aggregative behavior on growth, we measured the growth rates of individual cells on xylose and xylan within the microfluidic growth chambers (Supplementary Fig. 7a, b). We found that the maximal growth rate of cells at the end of the experiment was statistically similar in xylan and xylose in the microfluidic growth chambers (Supplementary Fig. 7c). This finding is in stark contrast to the impaired growth of cells that was observed on xylan in well-mixed batch cultures (Fig. 1b–d) and indicates that growing in a spatially structured monolayer within the microfluidic chamber allows cells on xylan to compensate for the growth reduction observed in well-mixed systems. However, the paths to similar growth rates were distinct in the two carbon sources. The growth rate of individual cells showed a statistically significant positive correlation with the cells’ birth-time, *i.e*. the time at which a cell originated in a division event, in the xylan but not in the xylose environment (Supplementary Fig. 7a, b). This suggests that in xylan, cells that arise later in a lineage, and therefore experience a higher cell density, have higher growth rates than cells that are born earlier. We then explicitly tested the influence of cell density on the growth rate of cells. For this, we analyzed the relationship between the median growth rate of all cells in a chamber within a given time window and the total number of cells present in the chamber during that time window. This analysis revealed that in both the xylan and xylose environments, the median growth rate increased with the number of cells in the chamber (Fig. 3a, b). However, in xylan, the slope of change in growth rate with the number of cells was on average four times lower than that in xylose (Fig. 3c). This finding indicates that although growth rate increases with cell number in both xylan and xylose environments, the increase on xylan required a higher cell density compared to xylose. (Supplementary Fig. 7d), an outcome likely caused by the colonial growth mode.

This contribution of cell density to increasing growth rate appears to reach a plateau on xylan, and becomes negative at high density on xylose (Supplementary Fig. 7e). Analysis of growth rates as a function of cell density shows that growth rates on xylan plateau after reaching a population size of 40–110 cells, beyond which there is likely no benefit of additional cells being present, whereas on xylose an increase in cell number likely introduces competitive effects between cells. This effect of competition is a plausible reason why final cell density only ranges between 18–65 cells within the chambers on xylose (Fig. 3b). Finally, we also tested if higher inoculum densities would have a positive effect on the growth dynamics in well-mixed batch cultures. These experiments revealed that increasing the initial inoculum density strongly reduced the time to reach maximum population size for populations growing on xylan (Fig. 3d: slope: −6.78 h per 10 cell increase in inoculum density) compared to populations growing on xylose (Fig. 3d: slope: −3.41 h per 10 cell increase in inoculum density).

Since our findings indicate that rapid growth on xylan requires colony formation, a strain which is unable to form colonies and thus cannot benefit from an increase in local cell density should show a growth reduction on xylan in the microfluidic chambers. In order to test this, we grew cells of *C. crescentus* NA1000 on xylan or xylose in microfluidic devices. The NA1000 strain lacks the adhesive holdfast, which should reduce the surface attachment of cells. We found that *C. crescentus* NA1000 cells were unable to form colonies on xylan (Supplementary Fig. 8a, Supplementary Video 4). Instead NA1000 cells had near constant cell densities over time on both, xylose and xylan (Supplementary Fig. 8b, c, Supplementary Videos 4 and 5). This resulted in cells growing on xylan having a lower final growth rate and displaying morphological defects compared to cells growing on xylose (Supplementary Fig. 8a, d, Supplementary Videos 4 and 5). This finding is in stark contrast to *C. crescentus* CB15 cells growing on xylan, which achieve similar growth rates to CB15 cells growing on xylose (Supplementary Fig. 7c). These findings not only lend credence to our idea that cells growing in xylan benefit from the effect of increased cell density but also suggest that holdfast adhesins play a key role in colony formation on xylan.

In addition, we also tested if colony formation was a consequence of the polymer xylan’s biophysical properties rather than nutritional properties. First, we measured the viscosity of xylan and xylose solutions, and found water-like viscosity values for xylan and xylose solutions of respectively 0.88 ± 0.05 and 0.92 ± 0.06 (mean over shear range ± standard deviation) mPa.s. Second, we asked if the presence of xylan influenced cellular mobility. We found that this was not the case. Swarmer cells displayed similar swimming velocities (xylan: 28.10 ± 11.86 μm s^−1^, xylose: 23.49 ± 7.93 μm s^−1^; mean ± standard deviation) on both xylan and xylose (Supplementary Fig. 9a–c, Supplementary Videos 6 and 7). Third, we grew *C. crescentus* CB15 cells within microfluidic chambers in a mixture of both 0.05% xylan and 0.05% xylose. The expectation here was the cells growing on dual carbon sources should switch to solitary lifestyles since they no longer require an increase in cell density due to presence of xylose. We found that cells growing on both xylan and xylose mostly display solitary behaviors (Supplementary Fig. 10a, Supplementary Video 8) that do not result in substantially increases in the local cell density (Supplementary Fig. 10b). The maximal growth rate is achieved at a lower cell density (Supplementary Fig. 10c, d) and is comparable to the cell density required on xylose (Supplementary Fig. 10e). Overall, our findings suggest that cells engage in colonial growth modes in the presence of the polymer xylan in order to benefit from increased cell densities.

### Spatial associations resulting from aggregation increase cellular access to xylan degrading enzymes

Two contrasting mechanisms could potentially explain the benefit of spatial associations. The first is that individuals could benefit from enzymes secreted by cells nearby [5]. *C. crescentus* expresses a suite of xylanases, which degrade xylan [16]. Such enzymes and their breakdown products can be rapidly lost via diffusion [30, 31]. A solution for cells is to minimize these losses by staying in close proximities of each other [30], allowing them to benefit from each other’s degradative activities. An alternative mechanism is that xylanases are not produced in well-mixed conditions or their expression is limited by lower cellular density in such conditions. In order to test these explanations, we probed the degradative activity and localization of xylanase enzymes.

We grew cells in well-mixed conditions in the presence of xylan and/or xylose and quantified their xylanase activity using a chromogenic analog of xylan that produces a fluorescent signal upon degradation by xylanases. We found that cells growing on xylan had on average a 1.4-fold higher xylanase activity than cells that grew on xylose (Supplementary Fig. 11). We also measured xylanase activity of populations growing on xylan that were started from a higher initial cellular density (2 × 10^7^ c.f.u. ml^−1^) and found that degradative activities were similar (Supplementary Fig. 11) to that of populations which were initiated from a lower starting density (1 × 10^5^ c.f.u. ml^−1^). The xylanase activity of cells growing on both xylan and xylose was on average 1.6-fold higher than cells growing on xylan and 2.4-fold higher than cells on xylose (Supplementary Fig. 11). This finding indicates that xylose positively influences the xylanase activity of cells when present along with xylan. Our observations suggest that in well-mixed conditions, xylanases are produced and their activity is not limited by cell density. These results instead indicate that growth in well-mixed conditions is likely limited due to a loss of breakdown products to diffusion.

**Fig. 4:**
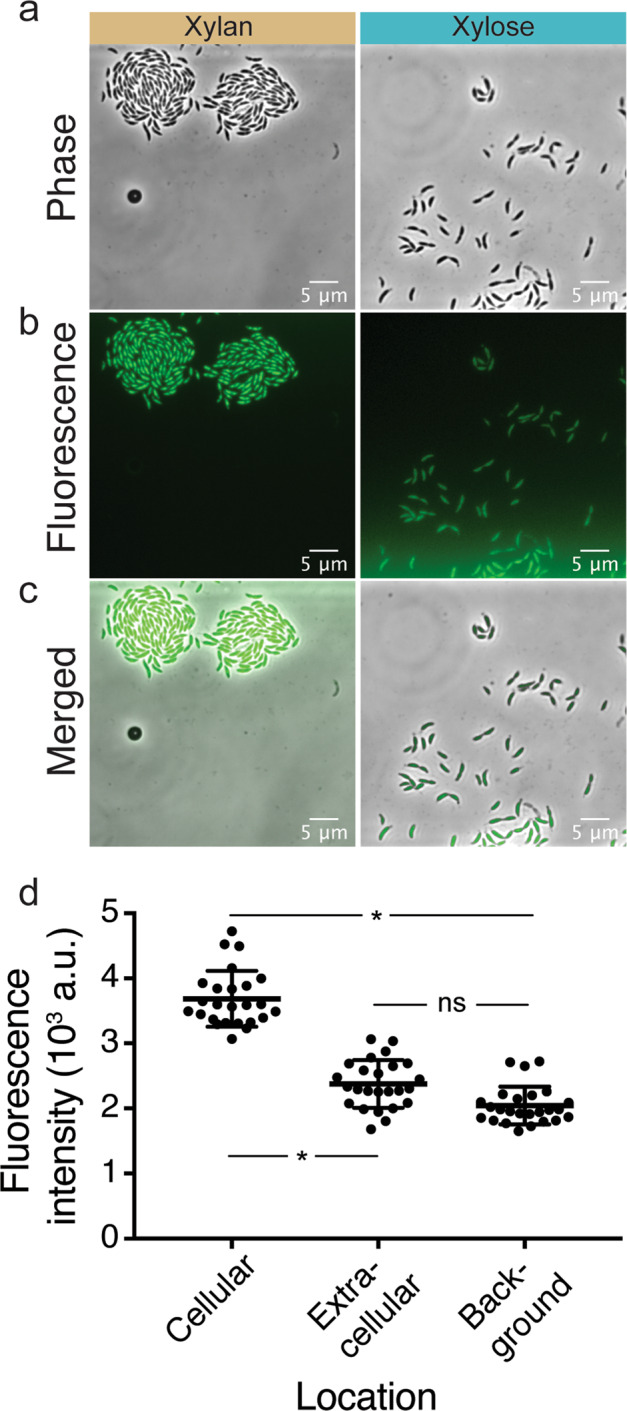
The activity of xylanase is localized on individual cells. **a**–**c** Xylanase activity (visualized using the degradation of a chromogenic analog of xylan) was present in cells growing on xylan (left panel) and negligible in cells growing on xylose (right panel). Representative (**a**) phase contrast, (**b**) fluorescence and (**c**) merged images of *C. crescentus* CB15 cells in one chamber that were grown for 18 h on xylose or xylan. Contrasts were adjusted to improve optical clarity but not for measurements in the images. **d** Mean fluorescence intensities (arbitrary units: a.u.) measured within cells, in their immediate vicinity (the extracellular region closest to the boundary of a cell) and in the background (a region without any cells). Points show the mean intensities for five cells and five corresponding extracellular and background regions each in five different microfluidic chambers, and horizontal lines show the mean and 95% CI. Asterisks and *ns* denote significant and non-significant differences between groups, respectively (independent samples *t*-test, FDR corrected *q* < 0.05, *n*_chambers_ = 5, *n*_cells*/*objects_ = 25).

We thus determined if spatial associations can result in increased access to enzymes or breakdown products by growing cells on xylan or xylose within microfluidic growth chambers and quantifying their xylanase activities. We found that cells growing on xylan had on average a 2.2-fold higher activity of xylanase than cells that grew on xylose (Supplementary Fig. 12a). This finding is qualitatively similar to the trend of xylanase activity in well-mixed conditions in the presence of xylan and xylose (Supplementary Fig. 11), suggesting that retention of breakdown products might positively influence xylanase activity. In addition, the fluorescent signal of xylanase activity localizes exclusively on the surface of individual cells. The intensity of the fluorescent signal in the immediate vicinity of cells was similar to the intensity in a background region with no cells (Fig. 4a–d). Similar to our finding regarding cell density in well-mixed conditions, the number of cells in a chamber did not influence the intensity of the fluorescent signal, suggesting that cell density does not affect xylanase activity (Supplementary Fig. 12b). These findings suggest that the enzyme is not released into the extracellular environment, but is instead surface-bound. Therefore, colonial growth reduces the spatial distances between cells, increasing a cell’s access to the degradation products of other cells and likely positively influences degradative activity.

### Changes in nutrient complexity and availability cause transitions in cellular behavior

**Fig. 5:**
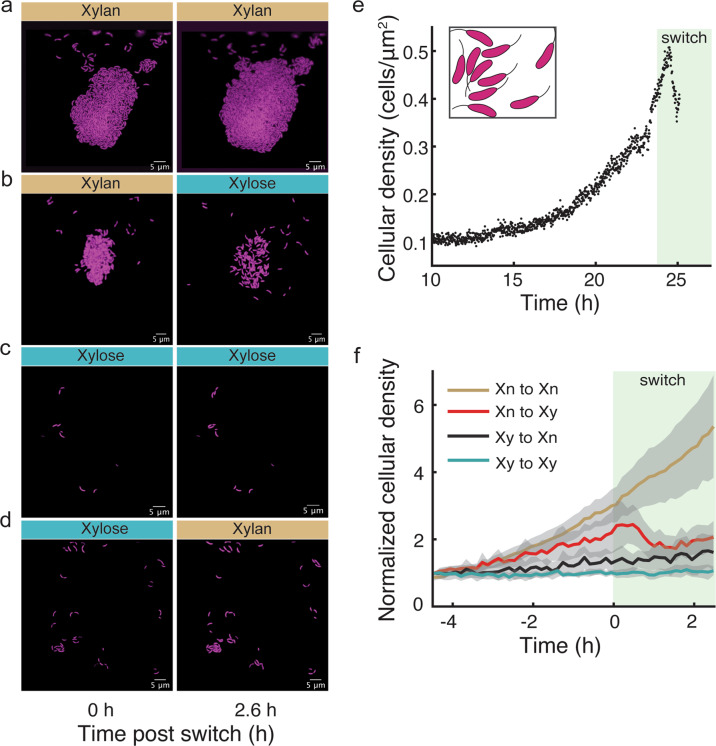
Cells transition between aggregative and solitary behaviors in response to change in nutrient complexity. **a**–**d** Time-lapse images of *C. crescentus* CB15 cells (labeled with constitutively expressed mKate2, false colored as magenta) within chambers exposed to constant conditions or switches in the complexity of the nutrients. **e** Cell density time series obtained from high frequency (1 frame per min) imaging of one chamber indicates that cell density increases while growing on xylan and starts declining ~40 min after the transition from xylan (Xn) to xylose (Xy). Switching time is indicated by the shaded background. Density is quantified as the number of cells in the area defined by the smallest rectangle encompassing each colony (illustrated by the figure inset), based on the (*x*,*y*) coordinates of the cells before the nutrient switch. See Supplementary Video 9 for a time-lapse of cells. **f** Cell density time series for different nutrient switches (at time 0 h; shaded background) based on images acquired every 8 min, each normalized to the colony density 4 h before the switch. Time series were averaged over *n*_*chambers*_ per condition (colored lines) and shown with the corresponding 95% confidence intervals (gray areas). Cells exposed to a xylan-to-xylan mock-switch continued their gradual increase in density (yellow, *n*_chambers_ = 10), while cells exposed to a xylose-to-xylose mock-switch did not change in average density (blue, *n*_chambers_ = 9, one chamber excluded because of very low initial density). Switching from xylan to xylose was followed by a decrease in density (red, *n*_chambers_ = 9), while little change in density was observed when switching from xylose to xylan (black, *n*_chambers_ = 9). See associated Supplementary Videos 10–12.

Given that cells form microcolonies on xylan but not on xylose, one would expect that environmental transitions in nutrient complexity from xylan to xylose would trigger a behavioral transition from colonial to solitary. In addition, an inverse switch from monomer to polymer should result in solitary cells forming microcolonies. To test for the ability of cells to tune their behavior in response to changes in their environment, we grew cells in microfluidic chambers on either xylan or xylose and then switched the growth environment to the alternative substrate. In line with our expectations, individuals rapidly dispersed when the environment changed from xylan to xylose (Fig. 5a–d, Supplementary Video 9). We quantified the colony density from images acquired at 1-min (Fig. 5e) or 8 min (Fig. 5f) intervals. This analysis revealed that cells disperse within 40 min of a nutrient switch from xylan to xylose (Fig. 5b, e, Supplementary Video 9). In contrast, a switch from xylose to xylan resulted in cells starting to form very small microcolonies (Fig. 5d) without drastic density changes in the 2.6 h following the environmental switch (Fig. 5f; Supplementary Video 10). Control switches from xylan to xylan and from xylose to xylose did not elicit behavioral changes (Fig. 5a, c, f; Supplementary Videos 11 and 12).

Behavioral transitions are also triggered by other situations in which the complex substrate xylan is no longer available. Subjecting microcolonies growing on xylan to transitions to a medium without any supplemented carbon source resulted in a two-fold reduction in cell density (Supplementary Fig. 13a). This observation indicates that the dispersal from a colony is a general response of *C. crescentus* to a shift in the environmental nutrient complexity (Supplementary Fig. 13a, Supplementary Video 13). When cells grown on xylan were subjected to a shift to a combination of xylan and xylose, a large variation was observed in the change of cellular density between replicate chambers (Supplementary Fig. 13b). Since xylose is produced due to xylan breakdown, cells likely are exposed to low levels of xylose while growing in colonies. Therefore, such a heterogenous response suggests that dispersal is likely the result of cells responding to multiple factors, which include the change in concentration of xylose and the presence or absence of xylan. In summary, our observations indicate that cells can flexibly modulate their behaviors in response to the presence or absence of a complex nutrient in the environment.

## Discussion

In natural ecosystems, cell aggregates or clumps are frequently observed when cells colonize and degrade particulate organic matter like chitin and alginate using chitinases and alginate lyases, respectively [10, 31–33], or degrade simpler polysaccharides like sucrose through the action of invertases [7]. Our study shows that such aggregative behavior can be a beneficial trait for bacteria growing on complex polysaccharides. An increase in local cell density results in an increase in the growth rate of cells. Since, the enzymatic activity is localized on individual cells, group formation likely allows cells to benefit from the activity of their immediate neighbors [20] through the exchange of breakdown products over small spatial distances and can potentially increase the rate of degradation of polymers [30, 33–35]. In addition, aggregation could be a strategy to exclude cells of other species or strains so that only kin benefit from breakdown products [30, 34].

That dispersal from aggregates and biofilms is triggered in response to changes in nutrient availability is well known [2, 4]. Our results showing departure from aggregates not only align well with previous findings but also provide novel insight into the role of nutrient complexity in driving behavioral transitions. It is known that bacterial cells can integrate information on nutrient availability and cell density to time their departure from biofilms [2]. Therefore, when the benefit of collectively degrading a resource no longer exists, cells are able to respond and engage in dispersal to solitary growth modes, which can serve multiple purposes. First, when simpler nutrients like monomers are present, cells can potentially reduce competition amongst individuals through reduced local densities [4, 17, 32]. The finding that an increased final cell density on the monomer xylose can reduce the final growth rate supports the existence of competition at high density. Second, cells can depart from aggregates to find and colonize new nutrient patches [4, 36]. The presence of monomers or absence of polymer in the environment could serve as a signal for cells that polymeric resources on particulate organic matter have reduced. In addition, cells can use chemotaxis systems in order to respond to such nutrient gradients in nature. It is known that *C. crescentus* possess multiple chemotaxis clusters which differentially regulate swimming behaviors towards nutrients like xylose or attachment to surfaces [37, 38]. Finally, since the formation of aggregates presumably requires the investment of cellular resources into attachment to surfaces or to other cells, dispersal into solitary modes might represent a cost-saving strategy for bacteria when aggregation no longer provides a benefit. Understanding how cells perceive environmental cues about nutrient complexity or changes in nutrient concentration and respond with appropriate behaviors will shed light on the regulatory pathways that govern such dynamic transitions.

The molecular mechanisms that drive collective growth behaviors and environment-induced transitions in bacteria have been widely studied, albeit in relatively non-natural environments [39, 40]. Our work uses a well-studied genetic model system to study growth on natural substrates and thus provides a basis to extend mechanistic studies in the context of the natural ecology of bacteria. It is known that cellular appendages like pili and holdfast adhesins allow *C. crescentus* cells to colonize surfaces [41, 42] and thus are likely involved in colony formation on xylan. In contrast, flagella allow cells to swim. Since, both flagellated swarmer and holdfast bearing stalked cells are present within colonies, it is likely that the motile swarmer cells drive the bulk of dispersal events. A systematic test of the role of such cellular structures on natural growth substrates will enable the unraveling of their ecological functions. Interestingly, it is known that the nutritional quality of the environment influences the activity of key regulators that modulate cell differentiation and adhesion in *C. crescentus* [19]. The loss of genes involved in in the biosynthesis of the adhesive holdfast can reduce growth and thus impose a fitness disadvantage in lake water [43], an observation in line with our findings with the holdfast deficient strain. In contrast, the disruption of surface attachment confers a fitness advantage when growing on simple sugars [43]. These findings imply that the ability to attach and form aggregates can be advantageous in natural ecosystems, where polymeric carbon substrates represent the dominant growth substrate [44]. We suggest that future work should focus on addressing the importance of processes that are involved in signaling within the cell and the regulation of these behaviors in mediating distinct phenotypic behaviors that help bacterial cells grow on ecologically relevant substrates.

## Conclusions

Our work uses a well-studied and ecologically relevant model system to directly control nutrient complexity in order to understand the effect on transitions between aggregative and solitary growth behaviors. Our results have important and direct implications for how the spatial associations and dynamics of individual cells influence the ecological and evolutionary properties of microbial populations in natural ecosystems. For example, cells live and divide in close proximity of each other within biofilms, which not only represent an important growth mode for bacteria in nature [45], but are also of relevance in industrial and medicinal applications. Finally, our findings elucidate the importance of a potentially ubiquitous strategy in bacterial populations, whereby individuals can transition between distinct behavioral states in response to fluctuating environments.

## Supplementary information

Supplementary information

Supplementary video 1

Supplementary video 2

Supplementary video 3

Supplementary video 4

Supplementary video 5

Supplementary video 6

Supplementary video 7

Supplementary video 8

Supplementary video 9

Supplementary video 10

Supplementary video 11

Supplementary video 12

Supplementary video 13

## Data Availability

All curated imaging datasets and source data for figures are deposited in the Zenodo repository (10.5281/zenodo.4603218).
