# Supplementary material for: Nutrient complexity triggers transitions between solitary and colonial growth in bacterial populations
Source: ISME J. 2021 Mar 17;15(9):2614–26. doi: 10.1038/s41396-021-00953-7 (PMC8397785; doi:10.1038/s41396-021-00953-7)
Supplement: Supplementary file 1 — Supplementary information [file 41396_2021_953_MOESM1_ESM.docx]

Supplementary Information for:

Nutrient complexity triggers transitions between solitary and colonial growth in bacterial populations

Running title: Bacterial cells tune behaviors to nutrient complexity

Glen G D’Souza^1,2*^, Vanessa R Povolo^1,2^, Johannes M Keegstra^3^, Roman Stocker^3^ and Martin Ackermann^1,2^

^1^Microbial Systems Ecology Group, Institute of Biogeochemistry and Pollutant Dynamics, Department of Environmental Systems Sciences, ETH-Zurich, 8006 Zurich, Switzerland

^2^Department of Environmental Microbiology, Eawag: Swiss Federal Institute of Aquatic Sciences, 8600 Duebendorf, Switzerland

^3^Institute of Environmental Engineering, Department of Civil, Environmental and Geomatic Engineering, ETH Zurich, 8093 Zurich, Switzerland

Corresponding author: Glen Gerald D’Souza

Email: glengeralddsouza@gmail.com

**This file includes:**

Supplementary Methods

Supplementary Figures 1 to 13

Supplementary Videos 1 to 13

Supplementary References

**Supplementary Methods**:

*Bacterial strains:*

Wildtype (WT) strain *Caulobacter crescentus* CB15 (ATCC 19089) was transformed with the plasmids containing either one of the two constructs pLac:mKate2 or pLac:Venus using electroporation as described previously (1). Chromosomal recombinants were selected using sucrose counter selection^1^ and tested for fluorescence expression.

*Growth and media:*

For experiments, a small volume (approximately 5μl) was first inoculated from frozen glycerol stocks into 3ml Peptone Yeast Extract (PYE) broth (2) in 15ml culture tubes and grown for 30 hours in a shaking incubator (220 rpm) at 30°C. The PYE recipe is based on the medium described in (ref.2) and consists of 0.2% tryptone (AppliChem, Germany), 0.1% yeast extract (Sigma Alrich, 70161), 0.8 mM magnesium sulfate (Sigma Aldrich, Switzerland), and 0.5 mM calcium chloride (Sigma Aldrich, Switzerland). These cultures were then subjected to a second round of growth in fresh 3ml PYE-B in culture tubes and grown for 16 hours in a shaking incubator (220 rpm) at 30 °C. Cells from these cultures were used for experiments in the corresponding M2 minimal salts medium. The recipe for M2 minimal salts medium was based on (ref.2) and consists of 6.1 mM Na_2_HPO_4_ , 3.9 mM KH_2_PO_4,_ 9.3 mM NH_4_Cl, 0.5 mM MgSO_4_, 10 μM FeSO_4_-EDTA chelate, 0.5 mM CaCl_2_ (Sigma Aldrich, Switzerland) with xylan or xylose as the sole carbon sources. Stock solutions (2% weight/volume) of xylan (Megazyme, Ireland) and xylose (Sigma Aldrich, Switzerland) were made in nanopure water and then filter sterilized using 0.40μm Surfactant Free Cellulose Acetate filters (Corning, USA). These were then used to constitute M2 (3) minimal medium to the desired condition.

*Kinetic growth assays:*

Growth kinetics in xylan or xylose media were measured in 96 well plates using a micro-well plate reader (Biotek). Briefly, 1 ml of cell suspension from a sub-culture in PYE was centrifuged (13000 rpm for 2mins) in a 2ml microfuge tube. The supernatant was discarded and the cell-pellet was subject to two rounds of washing with M2 minimal medium without any carbon source. The cell pellet was suspended in 500 μl of M2 minimal medium without carbon source and the optical density measured and adjusted to 0.1OD. 5μl of this OD adjusted cell suspension was used to seed wells containing 195 μl M2 minimal medium with the corresponding carbon source. Each experiment was replicated 4 times.

*Microfluidics:*

Microfluidics experiments were performed as described previously (2,4-6). Briefly, microfluidic devices consisted of chambers ranging from 15 x 60 to 120 μm and 0.56 μm in height and facing a feeding channel of 22 μm in height and 100 μm wide (Fig. S1). All acquisitions were conducted in chambers which were 60x60 μm. Polydimethylsiloxane elastomers (PDMS, Sylgard 184 Silicone Elastomer Kit, Dow Corning, USA) were prepared by mixing in a ratio of 1.5∶10 and poured on a dust-free wafer and then degassed in a vacuum desiccator for 45 minutes. This mix was then baked for one hour at 80°C to facilitate curing. PDMS chips of approximately 4 x 5 cm and 50mm in height were cut out with a scalpel and inlet holes for medium supply and outlet were punched (diameter of holes 0.75mm). These chips harboured six independent feeding channels (Supplementary Fig. 1). The chips were cleaned with H20, dried under pressurized air and were bound to round (50 mm diameter) to no. 1 glass coverslips (Menzel-Gläser, Germany) by treating them for 30 seconds at maximum power in a Plasma Cleaner (PDC-32G-2, Harrik Plasma, USA). Bonding between the glass coverslip and PDMS chip was stabilized by heating on a thermal plate at 100°C.

Cells from an overnight culture (16h) were concentrated (1ml into 10μl) by centrifugation at 10,000g for 1 min. The cells (1μl) were loaded into the inlets of a PDMS chip using a 10 μl pipette. Once a sufficient number of cells entered chambers, medium connections were initiated by connecting the tubing to the inlets. Media was contained in 50-ml syringes (Pic Solution) and flow was facilitated with 1, 6 or 8 channel syringe pumps (NE-300, NE-1600 or NE-1800, New Era Pump Systems, USA). Syringe pumps were interfaced with the PDMS chip inlets using a combination of 20-G needles (0.9 × 70 mm; Huberlab, Switzerland), larger tubing (Tygon microbore S54HL, ID 0.76 mm, OD 2.29 mm; Fisher Scientific, Switzerland) and small tubing (Adtek, ID 0.3 mm, OD 0.76 mm, UK). Small tubing was also used to interface the outlet with a waste bottle. To calculate swimming speeds, cells were inoculated into microfluidic chambers similar to the protocols described above.

*Time-lapse microscopy:*

Microscopy imaging was performed using either Olympus IX81or IX83 inverted microscope systems with automated stage controllers (Marzhauser Wetzlar, Germany), shutters, and laser-based autofocus systems (Olympus ZDC 1 and 2). Several positions/chambers were imaged in parallel on the same PDMS chip, and phase-contrast and fluorescent (mKate2 and/or Venus) images of every position were taken every 8min. Images were acquired using an UPLFLN 100× oil immersion objective (Olympus) and an ORCA-flash 4.0 v2 or v4 sCMOS cameras (Hamamatsu, Japan). For image acquisition, the CellSens 1.18 and higher software package (Olympus, Germany) were used. Fluorescent imaging was achieved using a X-Cite120 120Watt high pressure metal halide arc lamp (Lumen Dynamics, USA) along with TXRED, YFP and DAPI fluorescent filters (Chroma, USA). The microscopy units and PDMS chip was maintained at 30°C using the cellVivo microscope incubation system (Pecon GmbH, Germany) or the Cube incubation system (Life Imaging Services, Switzerland).

*Image analysis:*

Image processing was done in Matlab v 2017b or newer in combination with ilastik v1.2 and newer (7) and/or SuperSegger (8). Only fluorescent images were used for alignment, segmentation, tracking and linking. Images were cropped at the boundaries of each microfluidic chamber. Growth properties and spatial locations were directly derived from the downstream processing tools of SuperSegger (gateTool and superSeggerViewer) and ilastik (Tracking plugin). Spatial distances between cells (Fig. 2c and Supplemental Fig. 2) were computed from segmentation data and using the R package *spatstat* (9). Lineage trees were reconstructed using a custom R script in combination with the *rgl* package in R. Using the cellular coordinates obtained from segmentation, spatial densities in each chamber before and after switch (Fig. 5b and c; Supplemental Fig. 9) were computed by dividing the number of bacteria by the area of the smallest rectangle encompassing the cells. Single solitary cells far away from the main colony, usually located at the chamber edge, were excluded from the analysis. Time series were aligned to the time of switch and normalized by the density approximately 4 hours before the switch.

*Xylanase staining:*

*C.crescentus* CB15 WT populations (with starting densities of 10^5^ or 10^8^ cfu ml^-1^ were grown in 100ml Erlenmeyer flasks with 10ml M2 medium containing either xylan, xylose or both, for 36 hours. 1ml of the culture was harvested, the absorbance (OD_600_) determined and then adjusted to 0.1 OD. Assays were performed with 50 μl of 0.1 OD adjusted culture, which was mixed with 50μl of solution containing 50μg/ml of fluorescent xylanase substrate in 1x Reaction buffer (EnzCheck Molecular Probes-Invitrogen, Germany). For the negative control, 50 μl of uninoculated medium with xylose was used. The samples were incubated at room temperature, protected from light, for 30 minutes. Fluorescence was measured in 96 well plates placed in a micro-well plate reader (Biotek) using excitation at ~360 nm and emission detection at ~460 nm. The experiment was replicated 4 times. The fluorescence values of uninoculated medium were subtracted from the fluorescence values of the samples for further analysis.

*C. crescentus* CB15 WT cells were grown within microfluidic chambers on M2 minimal medium with xylose and xylan for 16 hours. After this, flow was stopped and 1μl (50μg/ml) of fluorescent xylanase substrate in 1x Reaction buffer (EnzCheck Molecular Probes-Invitrogen, Germany) was introduced manually into channels by pipetting through the inlets. The PDMS chip was incubated for 30 mins for optimal staining and for the dye to diffuse into the channels followed by phase contrast and fluorescent imaging (DAPI filter, Excitation: 358nm and Emission: 455nm) to determine activity.

*Datasets and statistical analysis:*

Pre-existing non-linear regression models in GraphPad Prism v 8.0 (GraphPad Software, USA) were applied to determine relationships between independent measures such as: number of cells versus time (Fig. 2c), growth rate versus cell birth time (Supplemental Fig. 7, Supplemental Fig. 10), growth rate versus number of cells (Fig. 3a and b), and times to reach half maximum optical density versus initial cell density (Fig. 3d). The best regression model that fits the data was selected based on the highest *R*^2^ or eta^2^ value. Detailed equations for non-linear regression models are shown below:

**1. Number of cells versus time (Fig. 2c)**

**Y=Y_0_*exp(k*X)** ; where

Y0 is the Y (cell count) value when X (time) is zero.

K is the rate constant (h^-1^).

Doubling-time (h) is computed as ln(2)/K

**2. Growth rate versus number of cells (Fig 3a and b)**

**Y=Y_0_ + (Plateau-Y_0_)*(1-exp(-K*x))** ; where

Y0 is the Y (growth rate, h^-1^) value when X (number of cells) is zero.

Plateau is the Y (h^-1^) value at infinite times

K is the rate constant (h^-1^)

**3. Times to reach half maximum optical density versus initial cell density (Fig 3d).**

**Y=Y_intercept_ + Slope*log(X) ;** where

X is the inoculum size (c.f.u. ml^-1^)

Slope is the change in Y (time to half maximum OD, h) when the log(X) changes by 1.0 (so X changes by a factor of 10).

Y_intercept_ is the Y value when log(X) equals 0.0

**Supplementary Figures**:

*
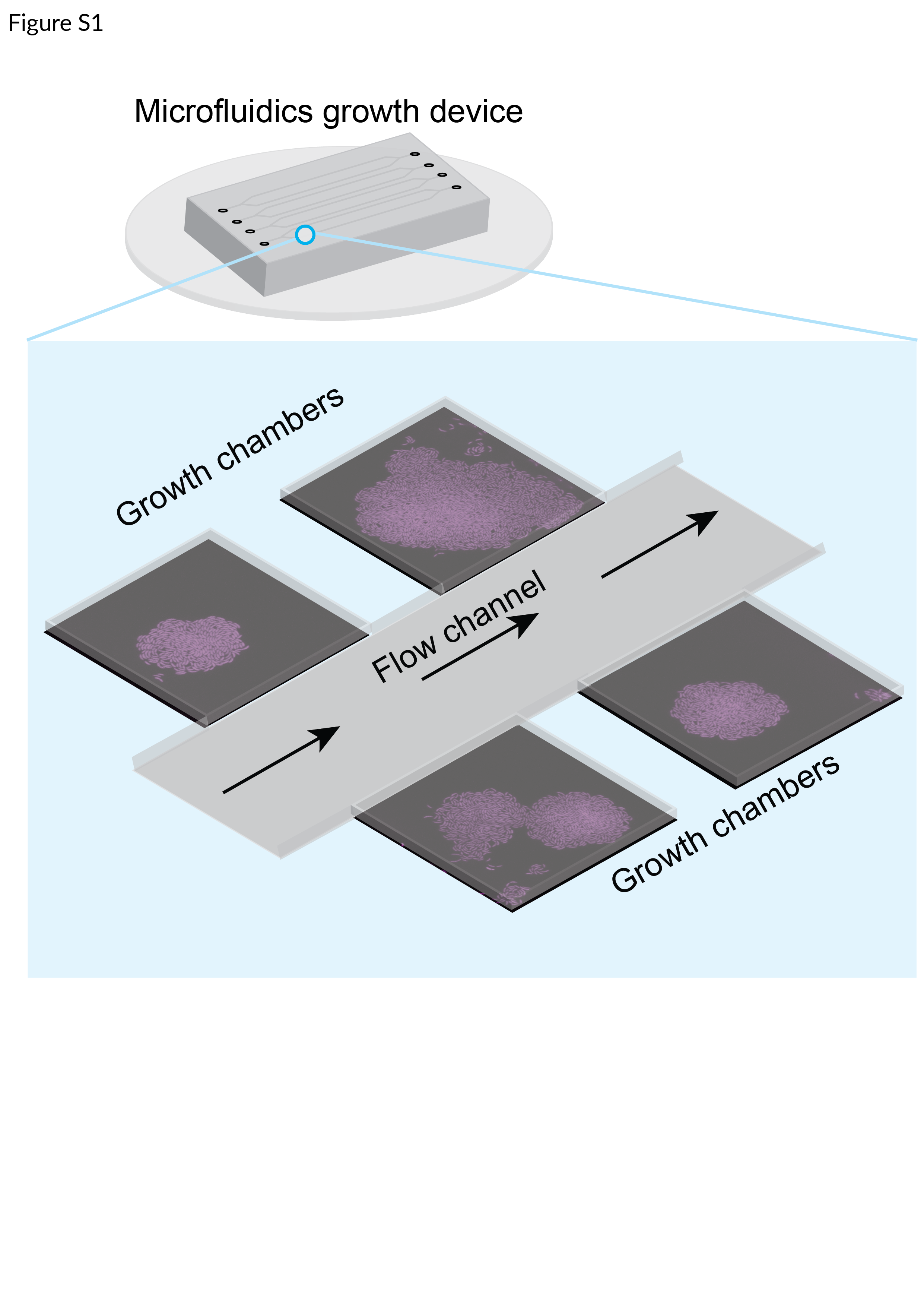
*

**Supplemental Figure 1**: **Design of microfluidic growth device and layout of the growth chambers.** Microfluidic devices were made out of Polydimethylsiloxane elastomers (PDMS) plasma bonded to a glass coverslip. Image acquisitions were conducted in chambers which were 60x60x0.56 μm and faced a nutrient feeding channel of 22 μm in height and 100 μm wide. Shown are snapshots of cellular aggregates at a representative time point in the acquisition process on xylan as the growth substrate.

**
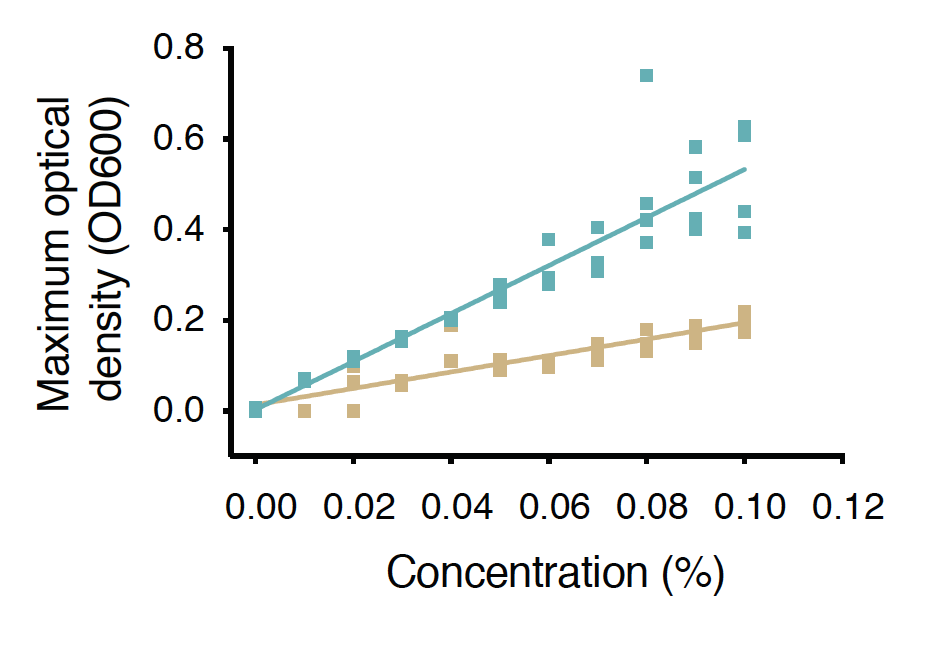
**

**Supplemental Figure 2**: **Maximum population size of the populations (measured as optical density at 600nm) attained by *C. crescentus* populations**. Squares indicate the measurements for each biological replicate (n=4) whereas the lines indicate the linear regression (yellow: xylan and blue: xylose). Cells generally achieve higher growth rates (h^-1^) on xylose as compared to xylan. Maximum optical density on xylose is generally higher compared to populations on xylan and increase in optical density with concentration is higher as compared to xylan (independent samples t-test, P<0.001, R^2^=0.28, n=4; linear regression model: xylose: R^2^=0.86, slope=5.3, P<0.001; xylan: R^2^=0.72, slope=1.8, P<0.001).


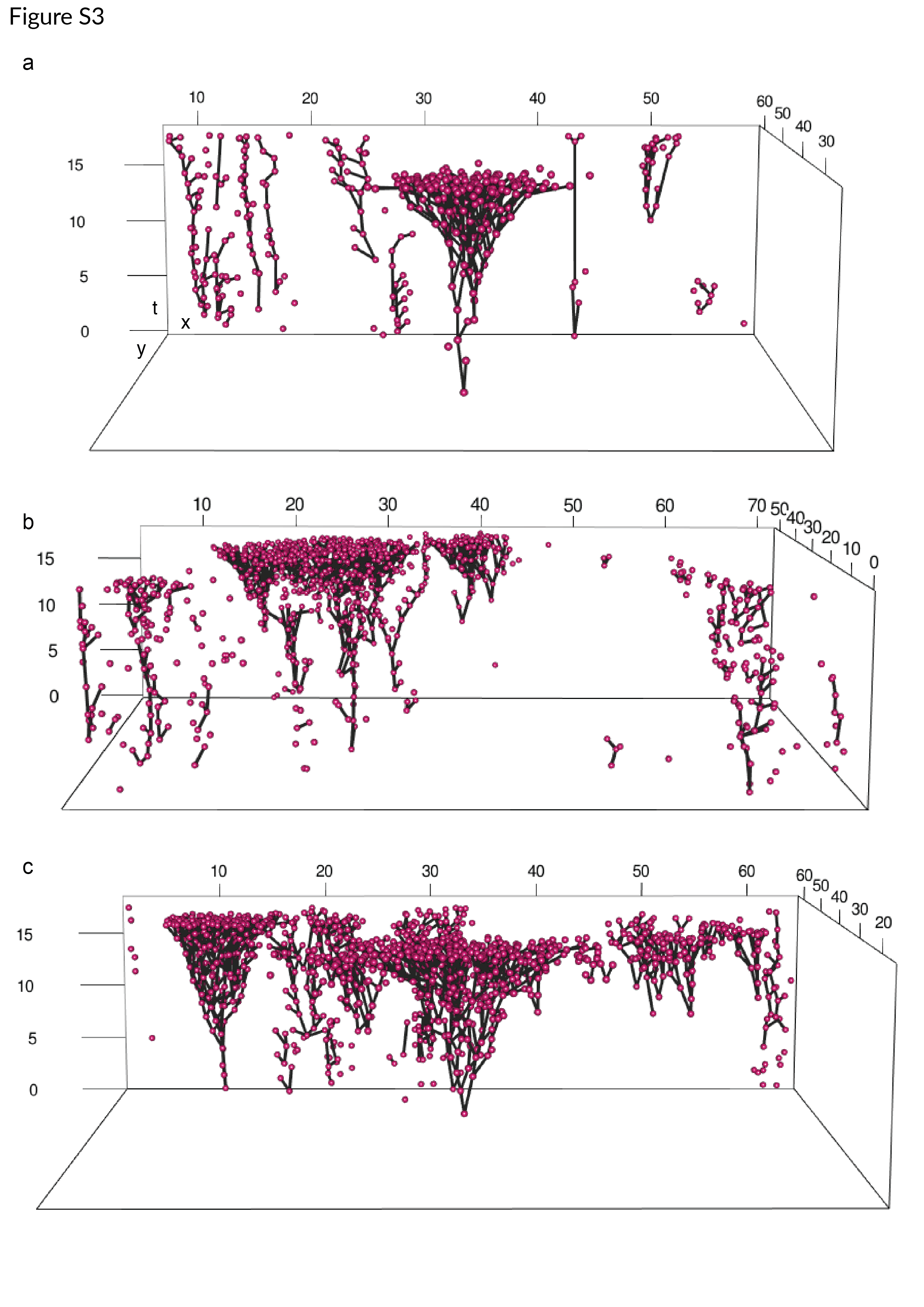


**Supplemental Figure 3. Cells form clonal collectives in xylan.** Lineage trees reconstructed from time-lapse images of cells within three chambers (**a**, **b** and **c**) on xylan. Cells (magenta spheres) are plotted as a function of their spatial location (horizontal plane) and time (vertical plane). Black lines connect cells which are related. Branching points in the lineage tree mark cell division events.

**
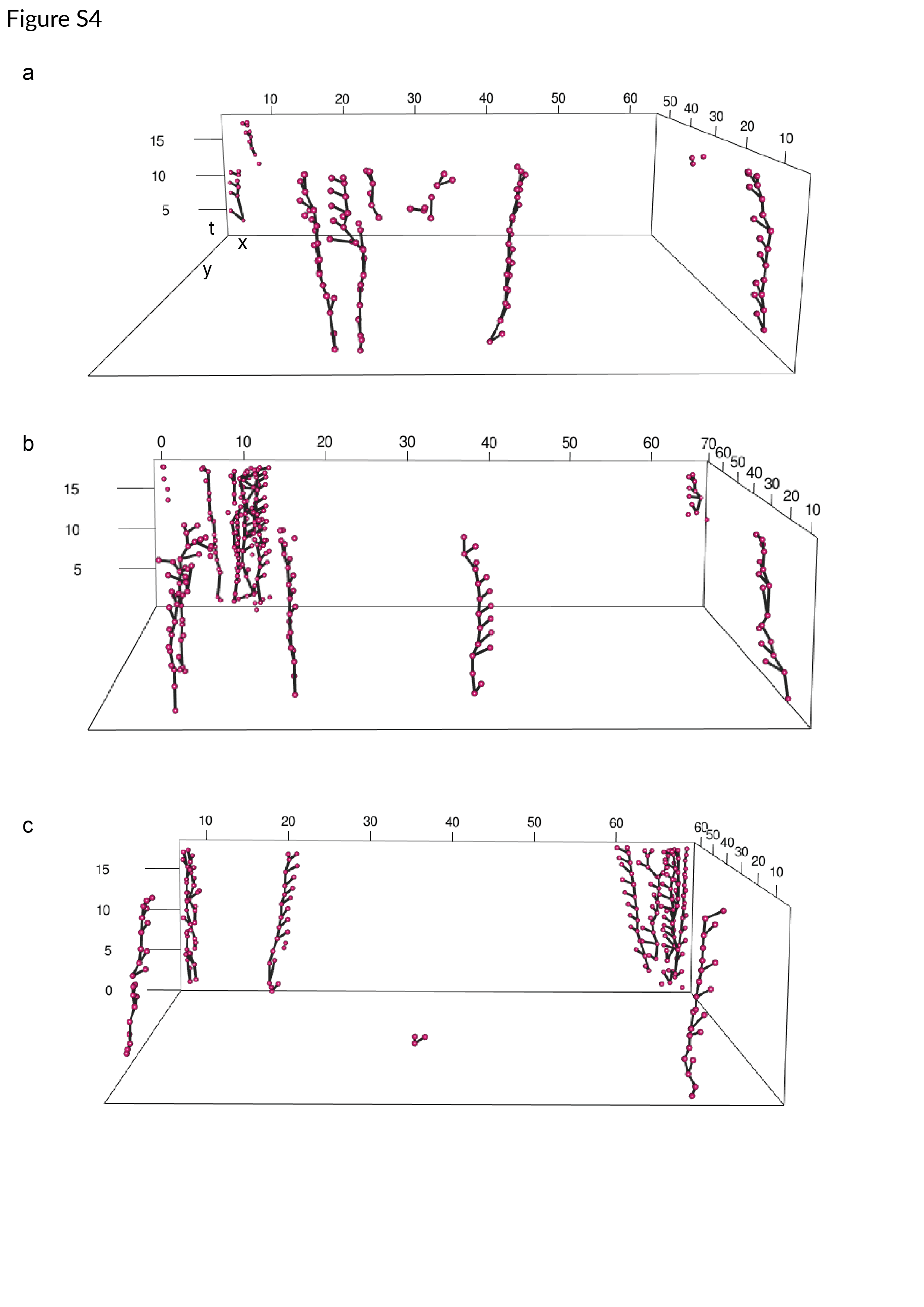
**

**Supplemental Figure 4. Cells exhibit solitary behaviours on xylose.** Lineage trees reconstructed from time-lapse images of cells within three chambers (**a**, **b** and **c**) on xylose. Cells (magenta spheres) are plotted as a function of their spatial location (horizontal plane) and time (vertical plane). Black lines connect cells which are related. Branching points in the lineage tree mark cell division events.

**
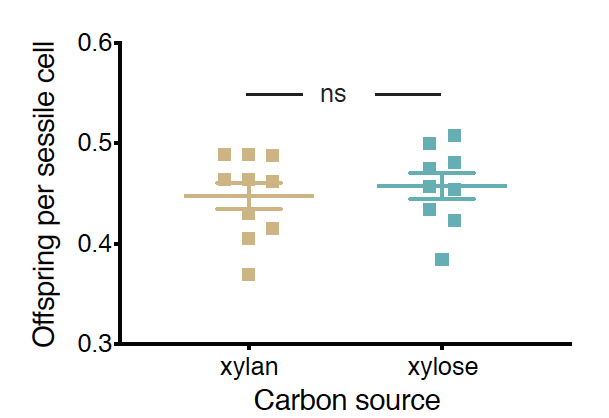
**

**Supplemental Figure 5: The propensity of sessile cells to produce offspring is similar in xylan and xylose.** For every cell that was born as a result of cell division, the probability it divided again was calculated based on lineage data obtained from segmentation and tracking. On average, 45% of cells growing on xylan within microfluidic chambers produce offspring while 46% of cells growing on xylose produce offspring. Points indicate measurements for each chamber on xylan (yellow) and xylose (blue), respectively. Horizontal lines indicate the mean and confidence intervals. Ratios are statistically similar in both environments (independent samples t-test, P>0.01, R^2^=0.01, *n_chambers_* = 9, ncells=2197 (xylose) and 11867 (xylan))


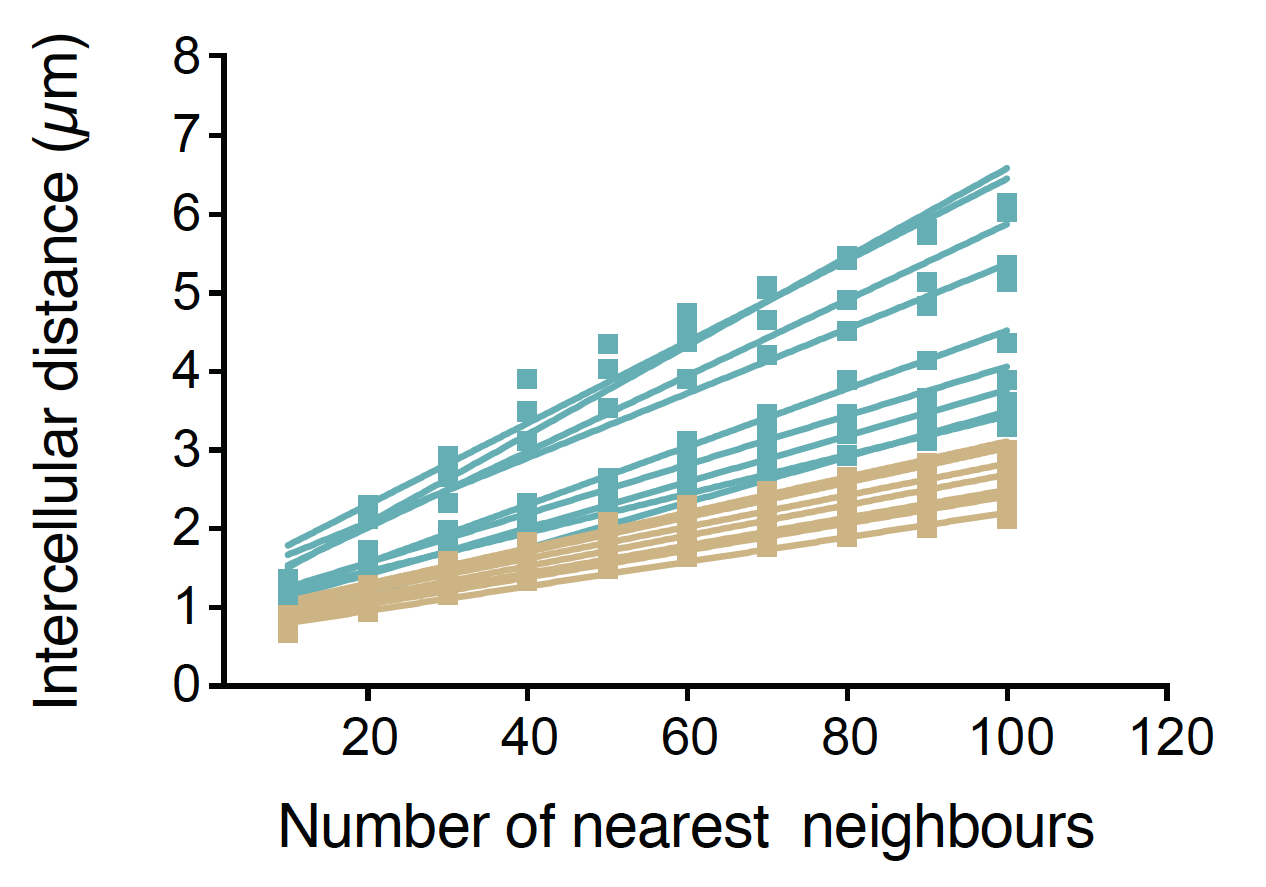


**Supplemental Figure 6: Increased intercellular distance when growing on xylose.** The difference in mean intercellular distance (μm) was small when 10 nearest neighbours were considered but increased when a larger number of neighbours was considered. Each circle indicates the mean intercellular distance between 10-100 nearest neighbours for all cells in each chamber (*n_chambers_*=9, *n_cells_*=2197 (xylose) and 11867 (xylan) and the lines indicate the linear regression model (yellow: xylan and blue: xylose; xylose: R^2^=0.92-0.98, slope=0.03-0.04, P<0.001; xylan: R^2^=0.95-0.98, slope=0.01-0.02, P<0.001).

**
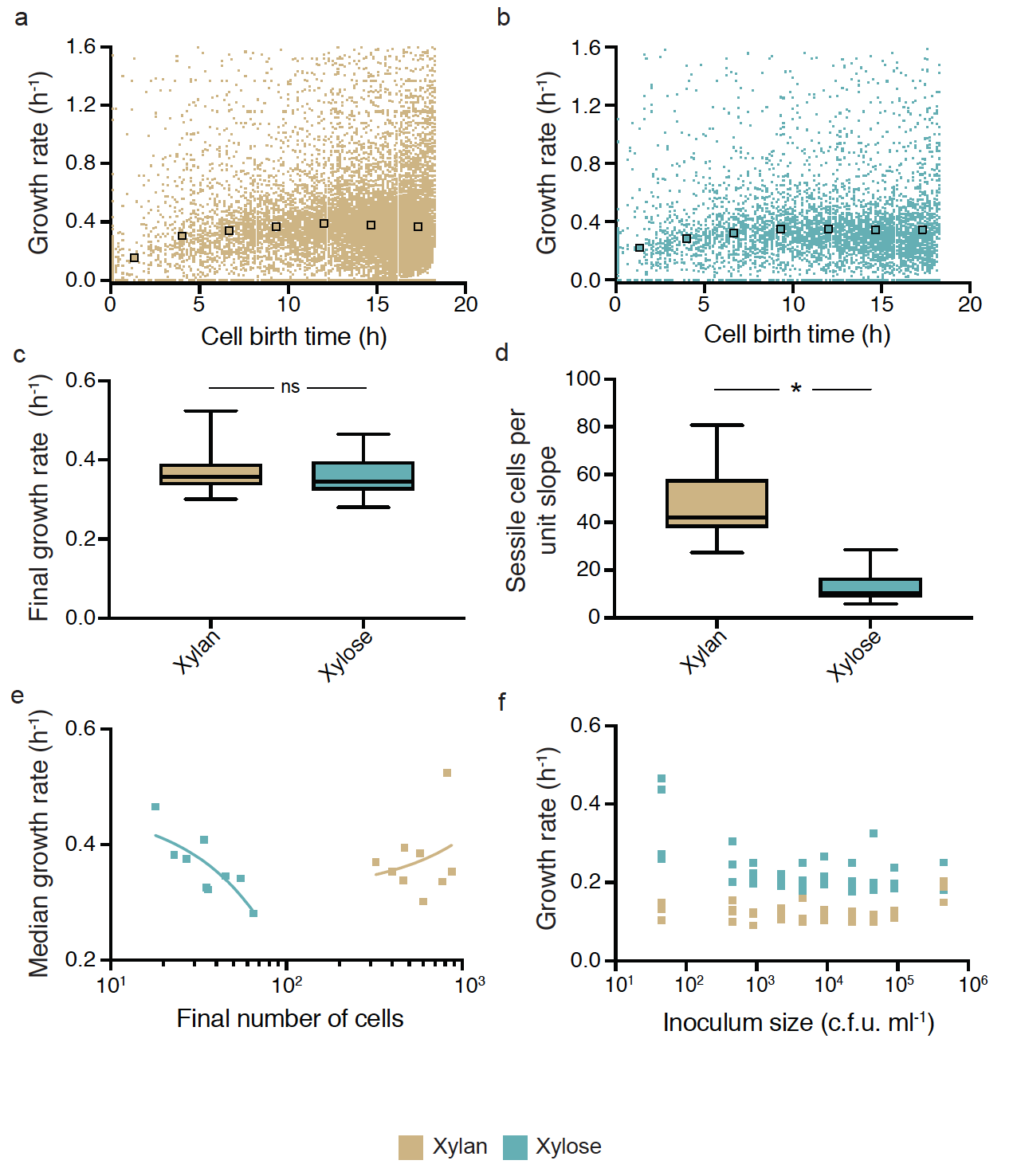
**

**Supplemental Figure 7: Growth rates of single cells on xylan and xylose environments.** Single-cell growth rates of all cells across all replicate chambers on (**a**) xylan and (**b**) xylose, as a function of their birth time. Each dot is a single cell (*n_cells_*= 11867 (xylan) and 2197 (xylose)). Squares indicate the median growth rates for several binned time points: 0-2.66 h, 2.67-5.33 h, 5.34-8 h, 8.1-10.66 h, 10.67-13.33 h, 13.34-16 h, 16.1-18.6 h). Growth rates of cells correlated significantly with birth time in xylan compared to xylose (Fisher-Z-Transformation to compare Spearman correlations, R_xylan_= 0.85 and R_xylose_= 0.71, Z=22.45, P<0.001 *n_chambers_*=9). (**c**) Median growth rates of cells that were present during the final birth time bin within chambers (16.1–18.6 h) did not differ significantly between cells in xylan and xylose (independent samples *t*-test, *P* = 0.65, R^2^ (eta squared) = 0.01, *n*_chambers_ = 9 for each treatment). (**d**) In xylan, on average a fourfold greater increase in the number of cells present in the chamber is required to change the slope of growth rate compared to that in xylose (independent samples *t*-test, *P* < 0.0001, *R*^2^ = 0.65, *n*_chambers_ = 9 for each treatment). In (**c**) and (**d**) box plots extend from the 25th to 75th percentiles (horizontal line) and whiskers indicate the 10^th^ (bottom) and 90^th^ (top) percentiles of the distribution. *Asterisks* indicate statistically significant differences between groups. (**e**) Cell density at the end of the experiment does not influence growth rate of cells in xylan (yellow squares: Spearman correlation, R=-0.08, P=0.84, *n_chambers_*=9; yellow line: linear regression, R^2^ = 0.08, P=0.45) but negatively influences growth rate of cells on xylose (blue squares: Spearman correlation, R=-0.81, P=0.01, *n_chambers_*=9; blue line: linear regression, R^2^ = 0.61, P=0.01). Each square indicates the median growth rates of cells that existed in the last 2h of the experiment (16.1 h-18 h) in each chamber. (**f**) Initial cell density (cfu ml^-1^) has a no influence on growth rate (h^-1^) in both xylan and xylose, for well-mixed *C. crescentus* CB 15 populations. Squares indicates the measurements for each biological replicate (Univariate ANOVA to compare inoculum densities, P>0.05, n_populations_=4).

**
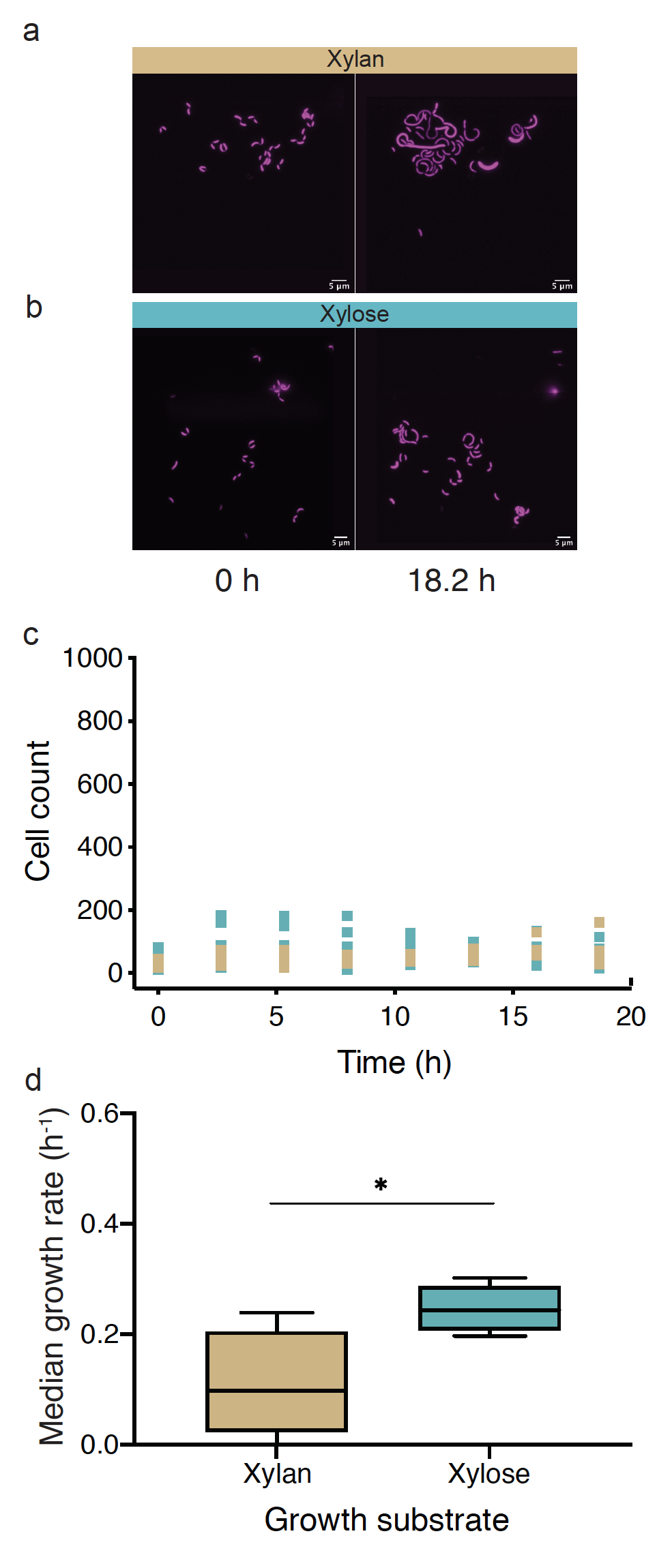
**

**Supplemental Figure 8. *C.crescentus* NA1000 strain that lacks holdfast is unable to form colonies on xylan** (**a** and **b**) Representative images of *C. crescentus* NA1000 cells (labelled with constitutively expressed mKate2, false colored as magenta) at different time points within the microfluidic growth chambers supplied with either 0.05% xylan (**a**) or 0.05% xylose (**b***)* as the sole source of carbon. (**c**) On both xylan (yellow) and xylose (blue), the number of sessile cells in the growth chamber remains nearly constant and are statistically similar across both treatments (Mann-Whitney Test, *P* > 0.05, *n*_chambers_ =6). Squares indicate the number of cells present at a given time point in each chamber (**d**) Median growth rates of cells that were present during the final birth time bin within chambers (16.1–18.6 h) was significantly different between NA1000 cells in xylan and xylose (Welch’s *t*-test, *P* = 0.06, R^2^ (eta squared) = 0.64, *n*_chambers_ = 6 for each treatment). Box plots extend from the 25th to 75th percentiles (horizontal line) and whiskers indicate the 10^th^ (bottom) and 90^th^ (top) percentiles of the distribution. The *asterisk* indicates statistically significant differences between groups. See associated Supplemental Videos 4 and 5 for time-lapse images of representative chambers.


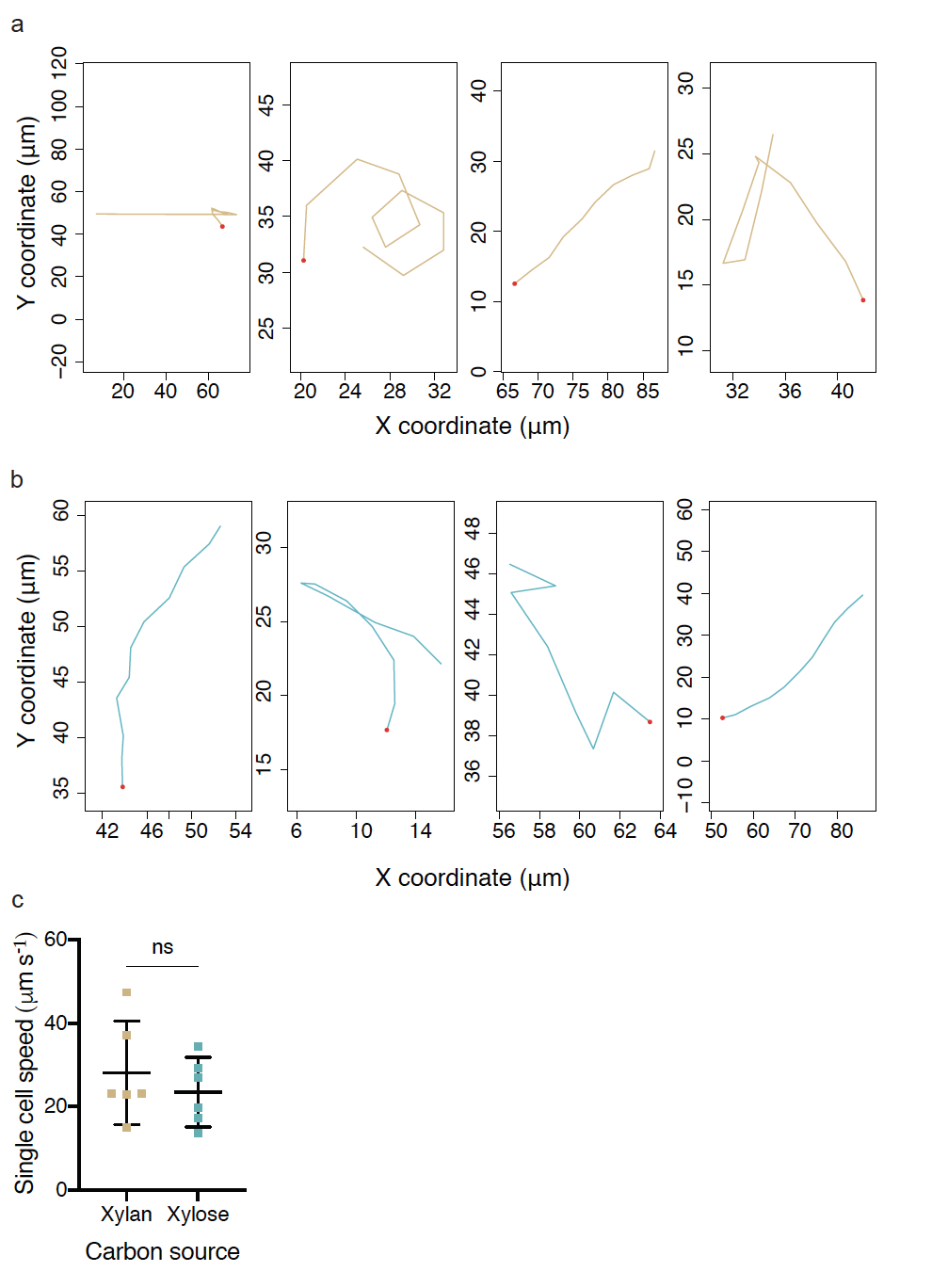


**Supplemental Figure 9: Mobility of swarmer cells is similar in xylan and xylose environments**. (**a** and **b**) Spatial trajectories of representative 8 swarmer cells mapped with a high frame rate (7.5 s^-1^) within microfluidic growth chambers in the presence of (a) xylan and (b) xylose, respectively. Red points mark the starting point of each trajectory. (c) Mean swimming speed of swarmer cells within microfluidic chambers over the course of a trajectory was statistically similar (denoted by ns) in xylan and xylose (Mann-Whitney Test, *P* = 0.58, U = 14, *n*_cells_ = 6 and *n*_chambers_ = 2 for each carbon source). Squares indicate mean swimming velocities (μm s^-1^) of individual cells, horizontal lines indicate the mean and whiskers indicate the 95% confidence intervals. See supplemental videos S6 and S7 for time-lapse images of swimming cells.

**
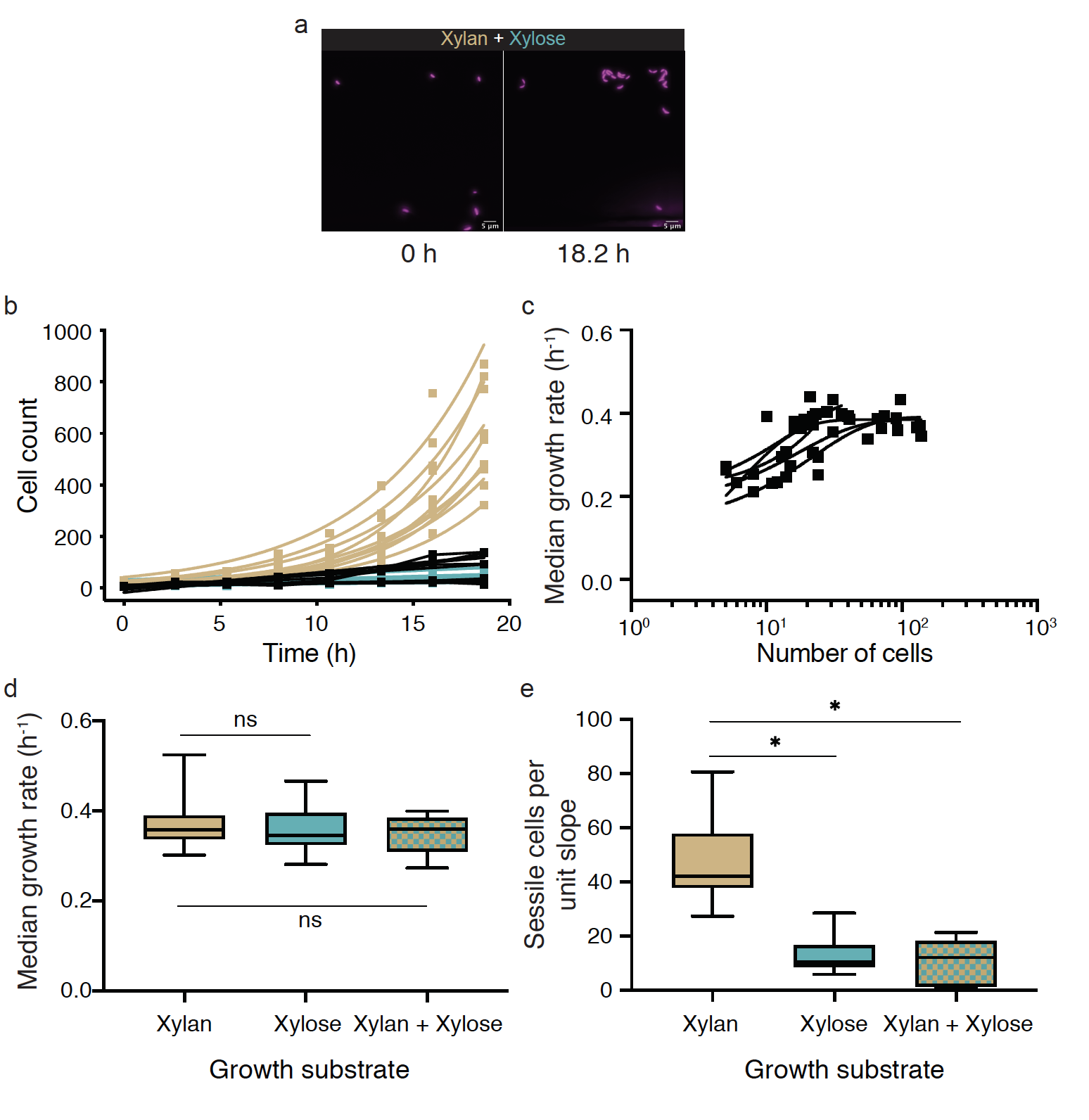
**

**Supplemental Figure 10. *C. crescentus* CB15 displays solitary behaviours in the presence of equal concentrations of xylan and xylose. (a**) Representative images of *C. crescentus* NA1000 cells (labelled with constitutively expressed mKate2, false coloured as magenta) at different time points within the microfluidic growth chambers supplied with both xylan (0.05%) and xylose (0.05%) as sources of carbon. (**b**) On xylan and xylose (black), the number of sessile cells in the growth chamber displays a moderate increase with time, whereas on xylan (yellow) it increases substantially and on xylose (blue) it remains nearly constant. Squares indicate the number of cells present at a given time point in each chamber (*n*_chambers_ = 5-9), with a linear or exponential regression line for each chamber (xylan and xylose, linear regression model, *R*^2^ = 0.33-0.92, slope = 1.422-7.58, *P* < 0.01; xylose, linear regression model, *R*^2^ = 0.69-0.92, slope = 1.22-3.27, *P* < 0.01; xylan, exponential growth model, *R*^2^ = 0.92-0.99, doubling time = 2.89-4.15h). Datasets for xylan and xylose are reproduced from Fig. 2. (**c**) After binning cells based on their birth times (bins: 0-2.66h, 2.67-5.33h, 5.34-8h, 8.1-10.66h, 10.67-13.33h, 13.34-16h, 16.1-18.6h) and hence the number of cells present during their growth, we determined which non-linear regression model can best predict (based on the *R*^2^ fit, see Methods and Supplemental Methods for detailed description) the relationship between median growth rate and cell number. Black squares represent data for a single bin from one chamber, and lines indicate the trajectory of growth rates for each chamber. In the presence of xylan and xylose, the relationship between median growth rate and the number of cells within a chamber was best explained by an exponential growth model (*n*_chambers_ = 5, *R*^2^ = 0.30–0.80). (**d**) Median growth rates of cells that were present during the final birth time bin within chambers (16.1–18.6 h) did not differ significantly between cells in xylan, xylose or xylan and xylan (Kruskall-Wallis test, *P* = 0.92, Kruskall-Wallis statistic = 0.14, *n*_chambers_ = 9 for xylan, 9 for xylose and 5 for xylan and xylose). (**e**) In xylan, on average a fourfold greater increase in the number of cells present in the chamber is required to change the slope of growth rate compared to that in xylose (independent samples *t*-test, *P* < 0.0001, *R*^2^ = 0.65, *n*_chambers_ = 9 for each treatment) or xylan and xylose (independent samples *t*-test, *P* < 0.001, *R*^2^ = 0.62, *n*_chambers_ = 5). In (**d**) and (**e**) box plots extend from the 25th to 75th percentiles (horizontal line) and whiskers indicate the 10^th^ (bottom) and 90^th^ (top) percentiles of the distribution. *Asterisks* and *ns* indicate statistically significant and non-significant differences between groups, respectively. Datasets for xylan and xylose are reproduced from Supplemental Fig. 7. See associated Supplemental Video 8 for time-lapse images of a representative chamber on xylan and xylose.


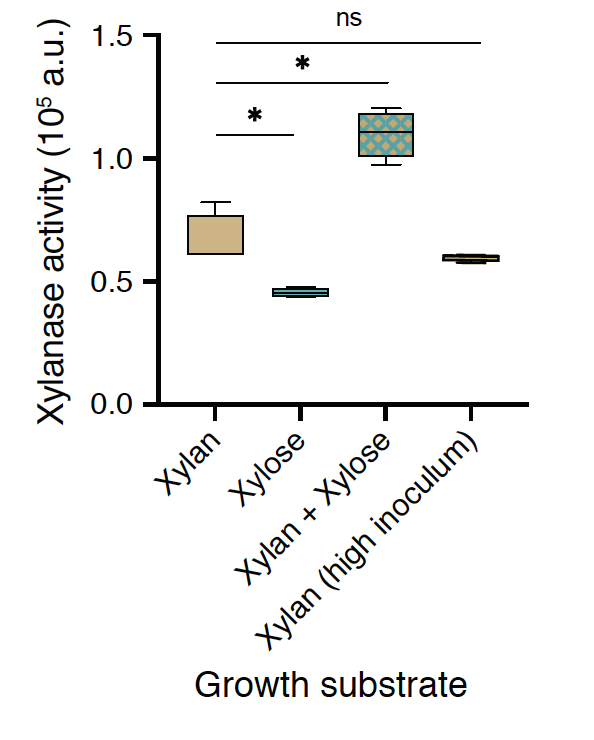


**Supplemental Figure 11: Xylanase activity of cells in well-mixed environments is higher during growth on xylan, positively influenced by xylose and is not influenced by cell density.** Cells growing on xylan display higher xylanase activity (measured using fluorescence intensities, arbitrary units: a.u.) than those on xylose (Mann-Whitney test: P=0.02, n_populations_ =4). Cells grown with xylan and xylose display higher xylanase activity than cells growing only on xylan (Mann-Whitney test: P=0.02, n_populations_ =4). Cells initiated from high initial density (2 x 10^7^ c.f.u ml^-1^) display similar xylanase activity to cells initiated from low density (1 x 10^5^ c.f.u. ml^-1^) on xylan (Mann-Whitney test: P=0.68, n_populations_ =4). Box plots extend from the 25th to 75th percentiles (horizontal line) and whiskers indicate the 10^th^ (bottom) and 90^th^ (top) percentiles of the distribution. *Asterisks* and *ns* indicate statistically significant and non-significant differences between groups, respectively.


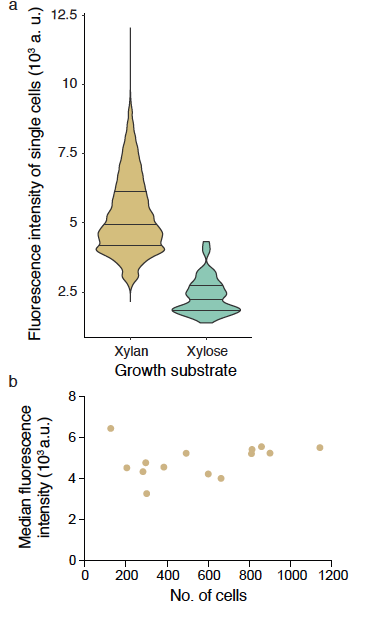


**Supplemental Figure 12: Xylanase activity within colonies is higher during growth on xylan and is not influenced by cell density.** (**a**) Cells growing on xylan display higher fluorescence intensities (arbitrary units: a.u.) than those on xylose (independent samples t-test: P>0.05, n_chambers_ =15 (xylan) and 10 (xylose), n_cells_=7874 (xylan) and 59 (xylose)). Violin plots show the distribution of single cell fluorescence intensities across all replicate chambers on xylan (yellow) or xylose (blue) and are scaled by width. Black horizontal lines indicate the quantiles (0.25, 0.5 and 0.75) of the distribution. (**b**) No correlation exists between number of cells in a collective and the fluorescence intensity of all cells in a chamber and the number of cells in a chamber (Spearman’s R= 0.23, P>0.05, n_chambers_ =15 (xylan) and 10 (xylose), n_cells_=7874 (xylan) and 59 (xylose)).

**
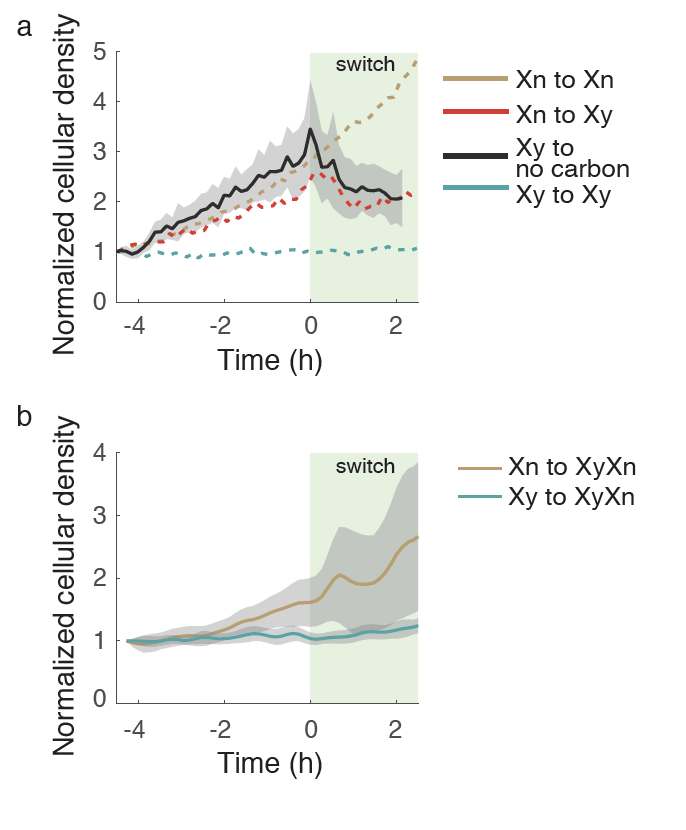
**

**Supplemental Figure 13:** **Dispersal from aggregates occurs when the environment is switched from xylan to non-carbon containing medium as well as a mixture of xylan and xylose.** (**a**) A switch from xylan to no-carbon medium results in dispersal of cells, as measured by a reduction in cellular density in the chamber (black line, n_chambers_ =5). The density change is similar to the change in colony density during a transition from xylan to xylose (red dashed line). Lines depict colony densities that are aligned at the time of switching (shaded green background) and normalized to the density 4 hours before the switch. The other environmental changes of Fig 5C are shown for reference as a yellow (xylan to xylan) and blue (xylose to xylose) lines. (**b**) Colony density time series, when switching from xylan to a mixture of xylan and xylose (yellow line, n_chambers_ =7) and xylose to a mixture of xylose and xylan (blue line, n_chambers_ =8). Time series were aligned and normalized as in panel A but with additional low-pass filtering with a moving average window of 24 minutes, In **a** and **b**, grey area indicates 95% CI based on standard error. Green background denotes the nutrient switch. See associated Supplemental Videos 9-13.

**Supplemental Videos**:

*Caulobacter crescentus* WT-mkate2 or *Caulobacter crescentus* WT-Venus cells growing in microfluidic growth chambers. Microscopy images were taken using phase contrast, TXRED or YFP channels every 1 or 8 minutes. Cells are false colored in magenta. Frame rate for all movies is 10 images per second except Supplemental Video 4 which has a frame rate of 40 per second. Scale bar is 5 microns

**Supplemental Video 1 (separate file).** Time-lapse of *C. crescentus* cells within a representative microfluidics chamber fed with xylan as the sole carbon source. Images were captured every 8 minutes.

**Supplemental Video 2 (separate file).** Time-lapse of *C. crescentus* cells within a representative microfluidics chamber fed with xylose as the sole carbon source. Images were captured every 8 minutes.

**Supplemental Video 3 (separate file).** Lineage development within *C. crescentus* aggregates within a representative microfluidics chamber fed with xylan as the sole carbon source. Cells related via divisional history to the same founder cell have the same colour. Cells whose divisional history cannot be tracked are show in green. Images were captured every 8 minutes.

**Supplemental Video 4 (separate file).** Time-lapse of *C. crescentus* NA1000 cells within a representative microfluidics chamber fed with xylan as the sole carbon source. Images were captured every 8 minutes.

**Supplemental Video 5 (separate file).** Time-lapse of *C. crescentus* NA1000 cells within a representative microfluidics chamber fed with xylose as the sole carbon source. Images were captured every 8 minutes.

**Supplemental Video 6 (separate file).** Time-lapse of *C. crescentus* cells within a representative microfluidics chamber fed with xylan. Images were captured with a high frame rate (7.5 frames s^-1^).

**Supplemental Video 7 (separate file).** Time-lapse of *C. crescentus* cells within a representative microfluidics chamber fed with xylose. Images were captured with a high frame rate (7.5 frames s^-1^).

**Supplemental Video 8 (separate file).** Time-lapse of *C. crescentus* cells within a representative microfluidics chamber fed with both xylan and xylose as carbon sources. Images were captured every 8 minutes.

**Supplemental Video 9 (separate file).**  Time-lapse of *C. crescentus* cells within a representative microfluidics chamber fed initially with xylan and then switched to xylose. Images were captured every minute.

**Supplemental Video 10 (separate file).** Time-lapse of *C. crescentus* cells within a representative microfluidics chamber fed initially with xylose and then switched to xylan. Images were captured every 8 minutes.

**Supplemental Video 11 (separate file).** Time-lapse of *C. crescentus* cells within a representative microfluidics chamber fed initially with xylan and then switched again to xylan. Images were captured every 8 minutes. Timing of the switch is indicated with +Xylan signage.

**Supplemental Video 12 (separate file).** Time-lapse of *C. crescentus* cells within a representative microfluidics chamber fed initially with xylose and then switched again to xylose. Images were captured every 8 minutes. Timing of the switch is indicated with +Xylose signage.

**Supplemental Video 13 (separate file).** Time-lapse of *C. crescentus* cells within a representative microfluidics chamber fed initially with xylan and then switched again to medium without any carbon source. Timing of the switch is indicated with +NoCarbon signage. Images were captured every 8 minutes.

**References:**

1. Persat, A., Stone, H.A., & Gitai, Z. The curved shape of Caulobacter crescentus enhances surface colonization in flow. *Nature Commun.* **5,** (2014).

2. Mathis, R. & Ackermann, M. Response of single bacterial cells to stress gives rise to complex history dependence at the population level. *Proc. Natl. Acad. Sci. USA* **113,** 4224–4229 (2016).

3. Hottes, A.K. et al*.* Transcriptional Profiling of Caulobacter crescentus during Growth on Complex and Minimal Media. *J. Bacteriol.* **186,** 1448–1461 (2004).

4. Dal Co, A., Ackermann, M. & van Vliet S. Metabolic activity affects the response of single cells to a nutrient switch in structured populations. *J. R. Soc. Interface* **16,** 20190182 (2019).

5. Dal Co, A., van Vliet, S. & Ackermann M. Emergent microscale gradients give rise to metabolic cross-feeding and antibiotic tolerance in clonal bacterial populations. *Philos. T. R. Soc. B.* **374,** 20190080 (2019).

6. Dal Co, A., van Vliet, S., Kiviet, D.J., Schlegel, S. and Ackermann, M. Short-range interactions govern the dynamics and functions of microbial communities. *Nat. Ecol. Evol.* **4,** 366–375 (2020).

7. Sommer, C., Straehle, C., Kothe, U. & Hamprecht, F.A. Ilastik: Interactive learning and segmentation toolkit in *2011 IEEE International Symposium on Biomedical Imaging: From Nano to Macro*, pp. 230–233 (2011).

8. Stylianidou, S., Brennan, C., Nissen, S.B., Kuwada, N.J. & Wiggins P.A. *SuperSegger* : robust image segmentation, analysis and lineage tracking of bacterial cells: Robust segmentation and analysis of bacteria. *Mol Microbiol* **102,** 690–700 (2016).

9. Baddeley, A., Holger. E. & Rolf, R in Turner Spatial point patterns: methodology and applications with R. CRC Press/Taylor & Francis Group (2016).
